# Optimizing a CRISPR-Cpf1-based genome engineering system for *Corynebacterium glutamicum*

**DOI:** 10.1186/s12934-019-1109-x

**Published:** 2019-03-25

**Authors:** Jiao Zhang, Fayu Yang, Yunpeng Yang, Yu Jiang, Yi-Xin Huo

**Affiliations:** 1 Key Laboratory of Molecular Medicine and Biotherapy, School of Life Sciences, Beijing Institute of Technology, No. 5 South Zhongguancun Street, Beijing, 100081 China; 2 UCLA Institute of Advancement (Suzhou), 10 Yueliangwan Road, Suzhou Industrial Park, Suzhou, 215123 China; 3 Key Laboratory of Synthetic Biology, Institute of Plant Physiology and Ecology, Shanghai Institutes for Biological Sciences, Chinese Academy of Sciences, Shanghai, 200032 China

**Keywords:** *Corynebacterium glutamicum*, CRISPR-Cpf1, PAM, crRNA, Linear template, Isobutyrate

## Abstract

**Background:**

*Corynebacterium glutamicum* is an important industrial strain for the production of a diverse range of chemicals. Cpf1 nucleases are highly specific and programmable, with efficiencies comparable to those of Cas9. Although the *Francisella novicida* (Fn) CRISPR-Cpf1 system has been adapted for genome editing in *C. glutamicum*, the editing efficiency is currently less than 15%, due to false positives caused by the poor targeting efficiency of the crRNA.

**Results:**

To address this limitation, a screening strategy was developed in this study to systematically evaluate crRNA targeting efficiency in *C. glutamicum*. We quantitatively examined various parameters of the *C. glutamicum* CRISPR-Cpf1 system, including the protospacer adjacent motif (PAM) sequence, the length of the spacer sequence, and the type of repair template. We found that the most efficient *C. glutamicum* crRNA contained a 5′-NYTV-3′ PAM and a 21 bp spacer sequence. Moreover, we observed that linear DNA could be used to repair double strand breaks.

**Conclusions:**

Here, we identified optimized PAM-related parameters for the CRISPR-Cpf1 system in *C. glutamicum*. Our study sheds light on the function of the FnCpf1 endonuclease and Cpf1-based genome editing. This optimized system, with higher editing efficiency, could be used to increase the production of bulk chemicals, such as isobutyrate, in *C. glutamicum*.

**Electronic supplementary material:**

The online version of this article (10.1186/s12934-019-1109-x) contains supplementary material, which is available to authorized users.

## Background

*Corynebacterium glutamicum* is a facultatively anaerobic Gram-positive asporogenic bacterium with short rod-like cells that is widely distributed in soil. Generally recognized as safe, *C. glutamicum* is non-pathogenic and does not produce endotoxins [1, 2]. It has been used in fermentation for over 50 years. Under anaerobic conditions, *C. glutamicum* can efficiently convert glucose to various amino acids [3, 4], organic acids [5], higher alcohols [6], and polymers [7]. Traditional genetic engineering strategies in *C. glutamicum* rely on a *SacB*-based suicide vector [8]. This process is time-consuming and inefficient, with a false-positive rate of 20–40% [8–11]. CRISPR-Cpf1 has attracted much attention in the field of genome editing because it is easy to manipulate and has a low off-target rate. However, gene deletion or insertion in *C. glutamicum* using CRISPR-Cpf1 reaches editing efficiencies of only 5–15% [12]. Hence, it is of great importance to improve the editing efficiency of CRISPR-Cpf1 in *C. glutamicum*.

Double-strand breaks (DSBs) introduced by the CRISPR-Cpf1 system are lethal to most bacteria, including *C. glutamicum* [13], which lacks a non-homologous end joining mechanism. Therefore, DSBs serve as a selection pressure to enrich mutations repaired using the artificial donor DNA templates in this organism [14–18]. Since CRISPR-Cpf1-induced DSBs are lethal in *C. glutamicum*, the targeting efficiencies of crRNA are reflected by survival rates. crRNAs with low targeting efficiencies cause a high rate of false positives during genome editing, which result in the survival of many wild-type cells within the population [17, 19–21].

**Fig. 1:**
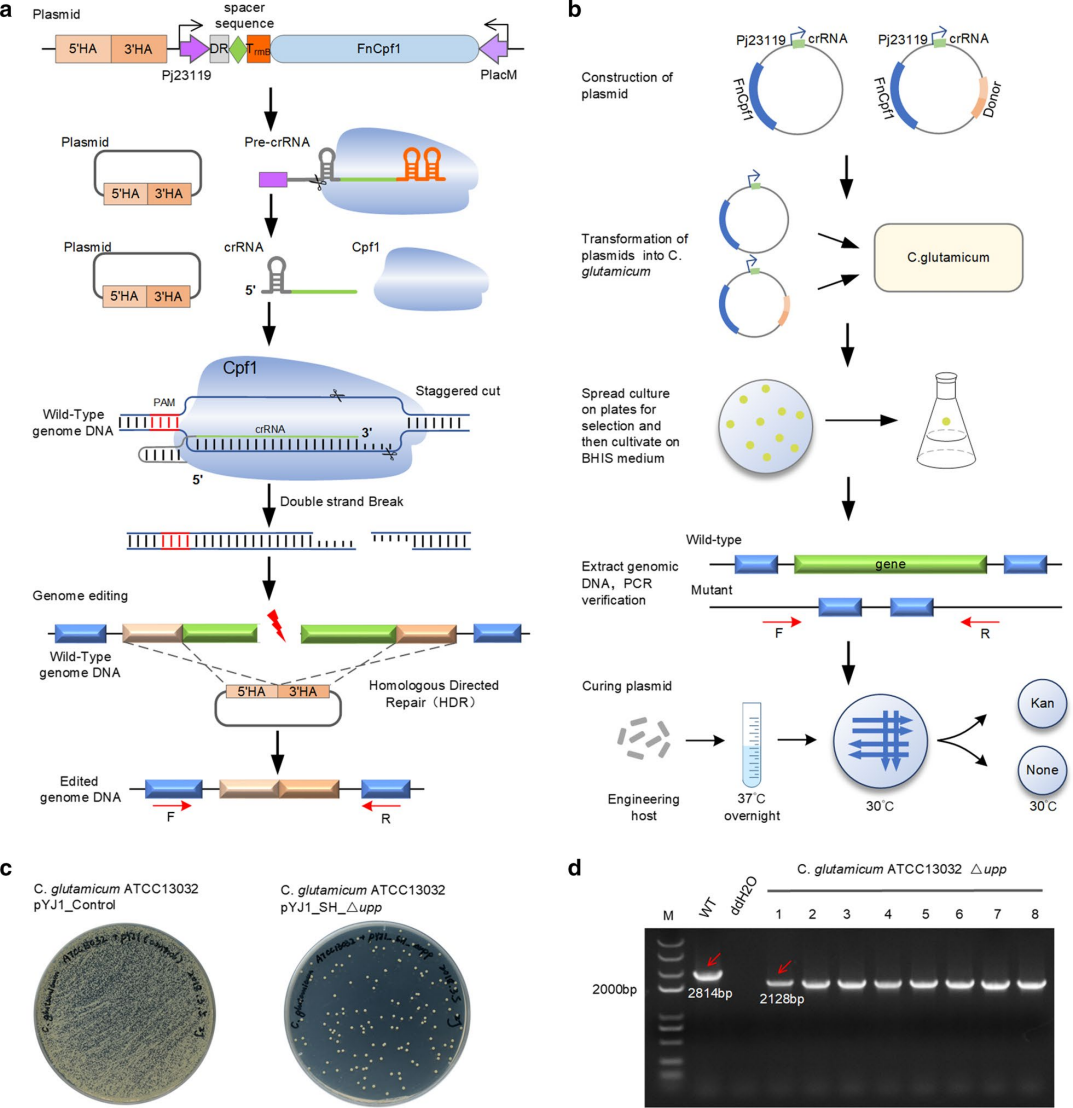
Schematic overview of the CRISPR–Cpf1-based system for iterative genome editing in *C. glutamicum*. **a** Schematic representation of genome editing using an all-in-one plasmid. The pre-crRNA is processed by Cpf1 into a mature crRNA, which then recognizes the target sequence and PAM sequence. The Cpf1-crRNA complex binds to the target site and induces a staggered cut to generate a double-strand break (DSB). The plasmid-borne template repairs the DSB through homologous recombination. The successfully edited colonies can be verified by PCR using the F/R primers located outside of the homologous arms. **b** A schematic representation of the procedure for CRISPR-Cpf1-assisted genome editing in *C. glutamicum*. A plasmid containing Cpf1, crRNA, and the repair template and another plasmid containing Cpf1 and crRNA are constructed. The two plasmids are then transformed into competent *C. glutamicum* cells and the cultures are spread on BHIS plates containing kanamycin. The single colonies are cultivated in complete medium to extract genomic DNA for PCR and DNA sequencing. The plasmid can be cured by overnight incubation in kanamycin-free medium at 37 °C and the correctly edited strain is then obtained by verifying antibiotic sensitivity. If necessary, the obtained strain can be used for the next round of editing. **c** The control (left) and edited (right) strain on the plates. The left plate is without 5-fluorouracil (5-FU) and the right plate is supplemented with 100 μM 5-FU. Under normal circumstances, the colonies on the right plate will all be positive. **d** PCR validation of *upp* gene deletion. The primers, F17/R17, used for verification, are located outside of the homologous arms. The amplified fragments are 2814 bp (wild-type) and 2128 bp (edited strain)

FnCpf1 requires a single crRNA and a thymine-rich PAM. crRNA contains a 19 bp direct repeat sequence and a 23–25 bp spacer sequence [22–25]. PAM is a 2–6 bp protospacer-adjacent DNA sequence (Fig. 1a), which is recognized by Cpf1 and plays an important role in both Cpf1 binding and cleavage [26]. In the presence of a PAM, the Cpf1-crRNA complex can bind the target DNA and induce DSBs. The cleavage site of Cpf1 is affected by the length of the spacer sequence. When the spacer length is greater than or equal to 20 bp, Cpf1 tends to cut the 18th nucleotide of the non-complementary strand and the 23rd nucleotide of the target DNA complementary strand. When the spacer length is less than 20 bp [27], Cpf1 tends to cut the 14th nucleotide of the non-complementary strand. The cohesive end introduced by Cpf1 facilitates homologous recombination (HR) [28]. Taken together, the PAM and the length of the spacer sequence are key determinants of the efficiency of Cpf1-dependent genome editing.

Editing efficiency is also influenced by the type of repair template. In *C. glutamicum*, the plasmid-borne dsDNA template can be utilized to repair the DSBs via HR [29]. Although linear DNA templates could be used to repair DSBs in the genomes of *C. elegans* [30], mouse, and human cells [31] with high efficiency, the repair of DSBs in *C. glutamicum* via linear DNA has, to the best of our knowledge, not been reported.

Here, we describe a CRISPR-Cpf1 based method for iterative genome editing and metabolic engineering of *C. glutamicum*. We established a lethal reporter system to verify the targeting efficiency of the crRNA and constructed a series of crRNA plasmids to characterize their cleavage activities in *C. glutamicum*. We optimized this system with respect to PAM sequence, the length of the spacer sequence, and the type of repair templates. Finally, the central metabolic pathways of *C. glutamicum* were modified by our genome editing method to improve isobutyrate production. The findings of this study will facilitate engineering of metabolic networks for the synthesis of value-added products using *C. glutamicum*.

## Results

### Establishing a CRISPR-Cpf1-mediated genome editing system in *C. glutamicum*

Our CRISPR-Cpf1 system contained three elements: a constitutive Cpf1 expression cassette, a crRNA expression cassette and a donor DNA template for repairing the DSBs introduced by the Cpf1-crRNA complex (Fig. 1a). As shown in Fig. 1b, each cycle of editing started with the transformation plasmid, pYJ1_S_∆gene, expressing crRNA or the plasmid, pYJ1_SH_∆gene, expressing the crRNA and donor DNA template in competent *C. glutamicum* cells. Unless repaired by HR in the presence of donor DNA templates, cells that took up a plasmid with functional crRNA were killed by Cpf1-mediated digestion. Eight colonies from a culture plate were picked and incubated in BHIS culture medium overnight and their genomic DNA was extracted individually. Gene knockout was verified by PCR, using primers that bind upstream of the 5′ homologous arm and downstream of the 3′ homologous arm. Mutants with the designed gene knockout were incubated at 37 °C in BHIS medium without antibiotics for plasmid curing and were then streaked on plates without antibiotics and grown at 30 °C (Fig. 1b). After confirming the loss of the plasmid, colonies were picked to prepare electrocompetent cells for the next round of editing.

To investigate the editing efficiencies of our system for gene deletion in *C. glutamicum*, the *upp* gene was chosen as the target gene, since its deletion leads to a 5-fluorouracil (5-FU)-resistant phenotype, which is convenient for screening [10]. We designed a crRNA targeting the *upp* gene and generated the plasmid, pYJ1_S_∆*upp*. The repair template was inserted into pYJ1_S_∆*upp* to create pYJ1_SH_∆*upp*. After engineering and plating, all 5-FU-resistant colonies on the plate were assumed to be *upp* knockouts. Eight colonies were confirmed to have the *upp* gene deleted by both PCR screening and sequencing, as shown in Fig. 1c, d. The efficiency of *upp* deletion was 100%. To further evaluate the efficiency of the system, we constructed pYJ1_SH_∆*crtYe/f* to delete the *crtYe/f* gene, since its deletion leads to a color change from yellow to red [32–34]. When the pYJ1_SH_∆*crtYe/f* plasmid was introduced into competent *C. glutamicum* cells, approximately 17% of single colonies were correctly edited, according to their color.

### Generation of a lethal reporter system and optimization of PAM sequence for the Cpf1-mediated genome editing system

**Fig. 2:**
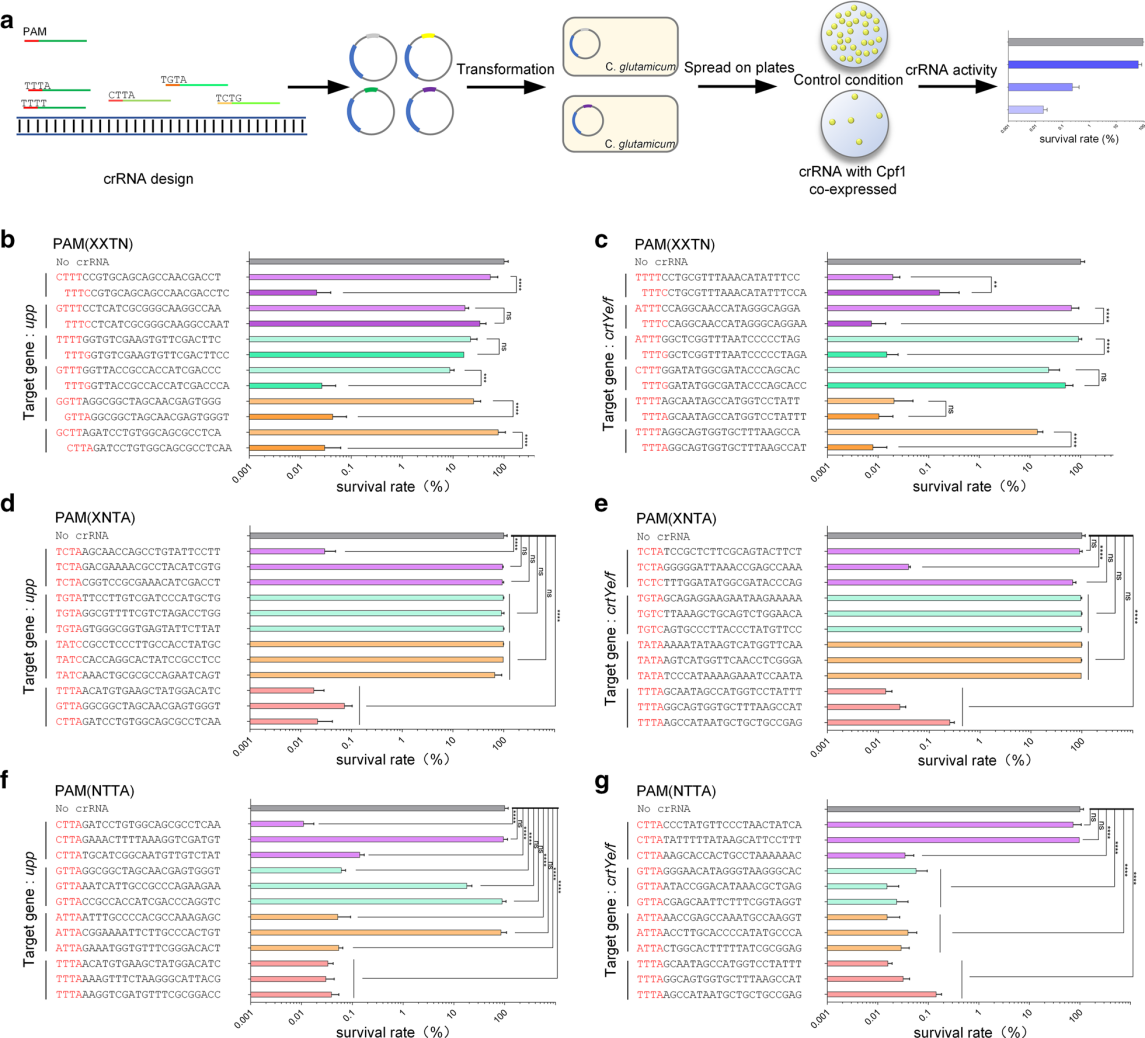
PAM sequence optimization for FnCpf1-mediated gene editing in *C. glutamicum*. **a** Illustration of the lethal reporter system for measuring Cpf1-mediated DNA cleavage efficiency in *C. glutamicum*. A series of crRNAs containing different PAM sequences are designed and inserted into the pYJ1 vector. Then, the *C. glutamicum* cells containing one of the pYJ1 derivatives are spread on plates in the presence or absence of antibiotics. The targeting efficiency of the crRNA can be calculated based on the number of colonies. **b**, **c** The effects of the last PAM sequence nucleotide (N) of 5′-XXTN-3′ on the efficiency of editing the *upp* (**b**) and *crtYe/f* (**c**) genes. All columns show survival rates (%), which are correlated with the targeting efficiency of the corresponding crRNA. **d**, **e** The effect of the second PAM sequence nucleotide (N) of 5′-XNTA-3′ on the efficiency of editing the *upp* (**d**) and *crtYe/f* (**e**) genes. X represents an unchanged nucleotide in the same set and N represents a changeable nucleotide. **f**, **g** The effect of the first PAM sequence nucleotide of 5′-NTTA-3′ on the efficiency of editing the *upp* (**f**) and *crtYe/f* (**g**) genes. Spacer and PAM sequences are shown in black and red, respectively. The gray column represents the control, the N in the purple column represents C, the N in the green column represents G, the N in the orange column represents A, and the N in the pink column represents T. The spacer sequences are also shown in Additional file 1: Table S2. Error bars; n = 3; data are analyzed using a paired-sample t-test. *P < 0.05, **P < 0.01, ***P < 0.001, ****P < 0.0001

Two genes (*upp* and *crtYe/f*) were selected to verify the optimal PAM sequence of CRISPR-Cpf1, using a lethal reporter system. We systematically profiled the targeting efficiencies of crRNAs by co-expressing crRNA with Cpf1. Different crRNAs were inserted into the pYJ1 plasmid to construct a series of pYJ1_S_∆*upp* or pYJ1_S_∆*crtYe/f* plasmids, which targeted different PAM sequences. The plasmid, pYJ1, without crRNA insertion, was used as the control. After transformation, the survival rate of cells in the presence of a specific crRNA was calculated using the formula shown in the “Methods” section (Fig. 2a). The efficiency of crRNA targeting of various PAM sequences was verified by the lethality rate of Cpf1-mediated gene editing.

The middle T is essential in the 5′-TTN-3′ PAM recognized by Cpf1 [25], and crRNAs with the PAM sequence, 5′-TTT-3′, have low activity in eukaryotes [35]. In order to investigate the influence of the final “T” in the PAM on the efficiency of CRISPR-Cpf1 in prokaryotes, we targeted chromosomal *upp* or *crtYe/f*. For each gene, six sets of crRNAs were designed to contain the four-nucleotide PAM sequence, 5′-XXTN-3′, where X represents an unchanged nucleotide in a given set and N represents any nucleotide. Each crRNA set contained two crRNAs, most of which were unchanged at the first three nucleotides and differed at the fourth nucleotide in the PAM sequence. In the first and second sets, the fourth nucleotides were T and C, respectively. In the third and fourth sets, the fourth nucleotides were T and G, respectively. In the fifth and sixth sets, the fourth nucleotides were T and A, respectively. Among the spacer sequences of the two crRNAs within each crRNA set, only one nucleotide was different. crRNA spacer sequences are shown in Fig. 2b. All 12 crRNAs targeting *upp* and all 12 crRNAs targeting *crtYe/f* were tested in the first round of experiments.

When the last nucleotide of the PAM sequence was T, the survival rates of cells with crRNAs targeting *upp* were 53.49 ± 17.65%, 17.03 ± 2.50%, 21.86 ± 6.23%, 8.64 ± 1.48%, 25.18 ± 7.74%, and 76.69 ± 23.95%. When the last nucleotide of the PAM sequence was C, G, or A, the survival rates of cells with crRNAs targeting *upp* were 0.021 ± 0.015%, 33.53 ± 8.67%, 16.27 ± 1.58%, 0.02 ± 0.01%, 0.04 ± 0.03%, and 0.03 ± 0.02%. When the last nucleotide of the PAM sequence was T, the survival rates of cells with crRNAs targeting *crtYe/f* were 0.02 ± 0.005%, 65.7 ± 21.77%, 91.17 ± 11.62%, 23.28 ± 12.35%, 0.02 ± 0.02%, and 13.98 ± 3.25%. When the last nucleotide of the PAM sequence was C, G, or A, the survival rates of cells with crRNAs targeting *crtYe/f* were 0.16 ± 0.19%, 0.007 ± 0.005%, 0.014 ± 0.008%, 50.06 ± 17.05%, 0.01 ± 0.007%, and 0.007 ± 0.005% (Fig. 2b, c). These results showed that when the last nucleotide of PAM was T, the efficiencies of that crRNA were the lowest compared with other corresponding nucleotides. This was consistent with previous studies in human cells [35]. Therefore, we avoided using T as the last nucleotide of the PAM sequence. Furthermore, the highest efficiencies were observed for crRNAs when the last PAM sequence nucleotide was A or C. Therefore, subsequent experiments were carried out using the PAM sequences with A or C as the last nucleotide.

We then tested the role of the second nucleotide of the PAM sequence. We designed, assembled, and tested crRNAs with 5′-XCTA/C-3′, 5′-XGTA/C-3′, 5′-XATA/C-3′, and 5′-XTTA-3′ PAM sequences. The survival rates of cells with the 5′-XCTA/C-3′ PAM sequence targeting *upp* were 0.29 ± 0.0001%, 94.42 ± 0.02%, and 93.37 ± 0.05% and targeting *crtYe/f* were 89.86 ± 0.09%, 0.03 ± 0.0002%, and 66.05 ± 0.08%. The survival rates of cells with the 5′-XGTA/C-3′ PAM sequence targeting *upp* were 97.51 ± 0.02%, 89.81 ± 0.09%, and 98.21 ± 0.02% and targeting *crtYe/f* were 94.7 ± 0.03%, 97.69 ± 0.02%, and 96.84 ± 0.01%. The survival rates of cells with the 5′-XATA/C-3′ PAM sequence targeting *upp* were 98.59 ± 0.004%, 97.69 ± 0.02%, and 66.62 ± 0.2% and targeting *crtYe/f* were 99.11 ± 0.008%, 96.84 ± 0.02%, and 96.84 ± 0.01%. The survival rates of cells with the 5′-XTTA-3′ PAM sequence targeting *upp* were 0.01 ± 0.0008%, 0.07 ± 0.002%, and 0.02 ± 0.001% and targeting *crtYe/f* were 0.01 ± 0.0003%, 0.02 ± 0.0006%, and 0.2 ± 0.0004%. Meanwhile, the survival rate of control cells was 100 ± 13.48% (Fig. 2d, e). These results showed that when T or C was the second nucleotide in the PAM sequence, the crRNA had a relatively high efficiency.

We next sought to test the effect of changing the first nucleotide using the PAM sequences, 5′-CTTA-3′, 5′-GTTA-3′, 5′-ATTA-3′, and 5′-TTTA-3′ (Fig. 2f, g). When the first nucleotide of the PAM sequence was T, the survival rates of cells with crRNAs were as low as 0.032 ± 0.007%, 0.030 ± 0.01%, 0.038 ± 0.01%, 0.014 ± 0.002%, 0.015 ± 0.002%, and 0.031 ± 0.008%, indicating that the crRNA containing the PAM sequence, 5′-TTTA-3′, had the highest targeting efficiency. When the first nucleotide of the PAM sequence was C, the survival rates of cells with crRNAs were 94.06 ± 5.84%, 73.10 ± 6.68%, and 96.78 ± 4.23%, indicating that the crRNA had a low targeting efficiency when the PAM sequence was 5′-CTTA-3′.

Taken together, these data clearly showed that FnCpf1 required a PAM sequence defined as 5′-NYTV-3′, where N represents any nucleotide, Y represents T or C, and V represents A, C, or G. Among these sequences, 5′-TTTA/C-3′ was the preferred design.

### The effect of spacer sequence length on Cpf1-mediated genome editing

**Fig. 3:**
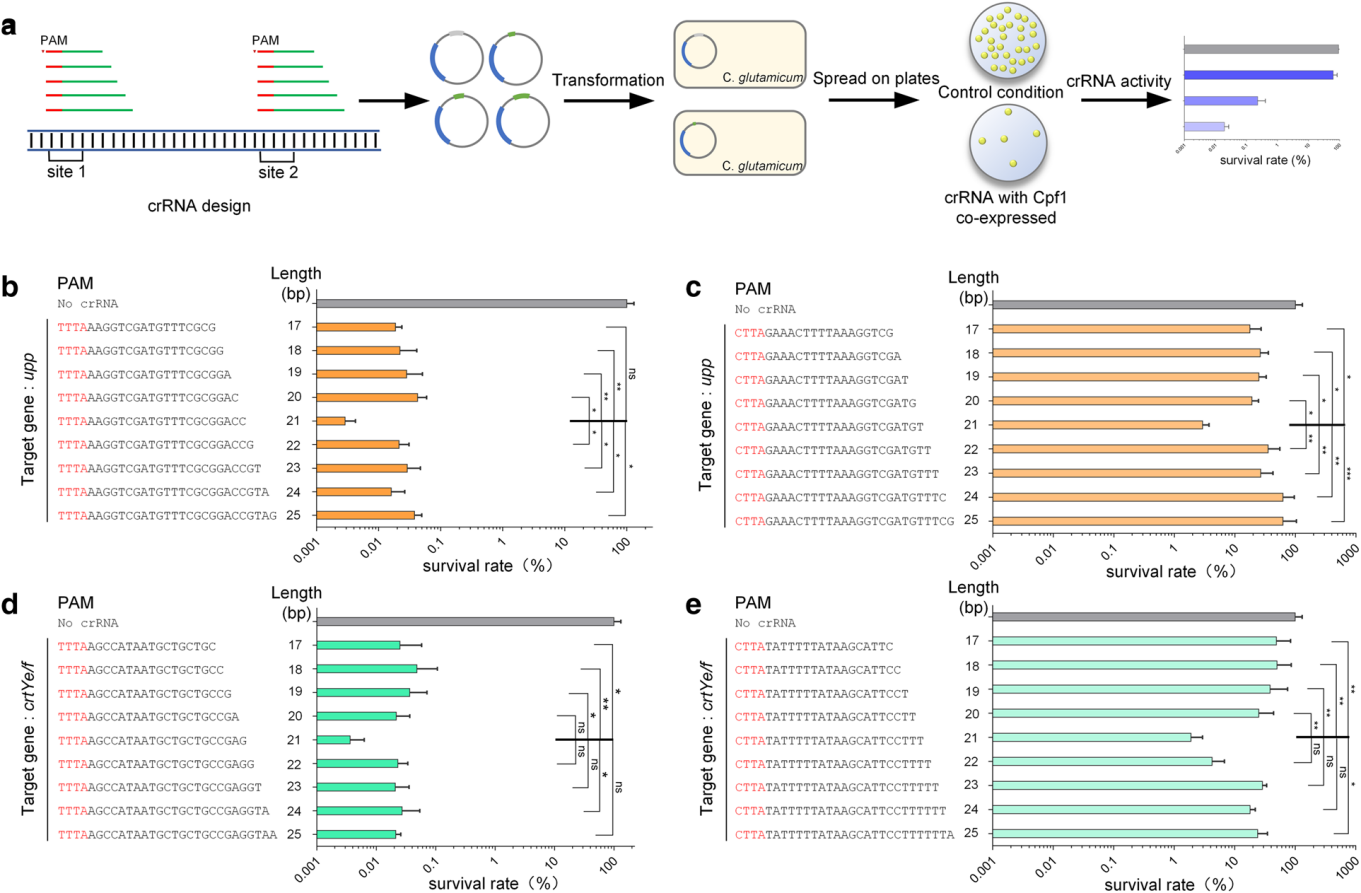
On-target gene editing efficiencies of crRNAs with spacer sequences of different lengths. **a** Schematic diagram of crRNA spacer sequence optimization. **b**–**e** The effect of the length and sequence of the crRNA spacer regions on editing efficiencies of different genes. crRNA targets *upp* using the PAM sequences, 5′-TTTA-3′ (**b**) or 5′-CTTA-3′ (**c**). crRNA targets *crtYe/f* using the PAM sequences, 5′-TTTA-3′ (**d**) or 5′-CTTA-3′ (**e**)

To determine the optimal length of the crRNA spacer sequence, 18 crRNAs targeting *C. glutamicum upp* and *crtYe/f* genes were constructed with 17–25 bp spacers (Fig. 3). A series of crRNAs, that could be functional in the presence of the preferred 5′-TTTA-3′ PAM sequence, were designed to cleave *upp* or *crtYe/f*. As shown in Fig. 3b, the survival rate of cells with the crRNA targeting *upp* was 0.003 ± 0.001% when the spacer length was 21 bp, while survival rates of cells with other crRNAs containing 17–20 bp or 22–25 bp spacer sequences were 0.016–0.042%. Similarly, the survival rate of cells with the crRNA targeting *crtYe/f* was 0.003 ± 0.002%, when the spacer length was 21 bp, while survival rates of cells with other crRNAs containing 17–20 bp or 22–25 bp spacer sequences were 0.021–0.048% (Fig. 3d).

To further confirm the effect of spacer sequence length on targeting efficiency, a series of crRNAs that could be functional in the presence of the relatively low-efficient 5′-CTTA-3′ PAM sequence, were designed to cleave *upp* or *crtYe/f*. The survival rate of cells with the *upp*-targeting crRNA containing a 21 bp spacer sequence was 2.91 ± 0.66%, while the survival rates of cells with other crRNAs containing 17–20 bp or 22–25 bp spacer sequences were much higher, ranging from 17.75 to 62.37% (Fig. 3c). Similarly, the survival rate of cells with the crRNA containing a 21 bp spacer sequence targeting *crtYe/f* was 1.89 ± 0.85%, while the survival rates of cells with the other crRNAs containing 17–20 bp or 22–25 bp spacer sequences ranged from 4.23 to 50.07% (Fig. 3e). These results showed that a 21 bp spacer sequence had the highest efficiency for gene editing using FnCpf1.

### The effect of repair template type on Cpf1-mediated genome editing

A DSB induced by CRISPR-Cpf1 in vivo can be repaired through HR in the presence of a plasmid-borne template [21, 36, 37]. In this study, we investigated whether another type of DNA could be used as a repair template. Two target genes, *upp* and *crtYe/f,* were chosen because *C. glutamicum* cells would undergo an obvious phenotype change if any of these two genes were deleted. Different repair templates were used to determine their effect on editing efficiencies. When *upp* was edited, the colonies became resistant to 5-FU and when *crtYe/f* was edited, the colonies turned red. The editing efficiency could be calculated by dividing the number of edited single colonies on the selection plate by the total number of colonies on the control plate.

**Fig. 4:**
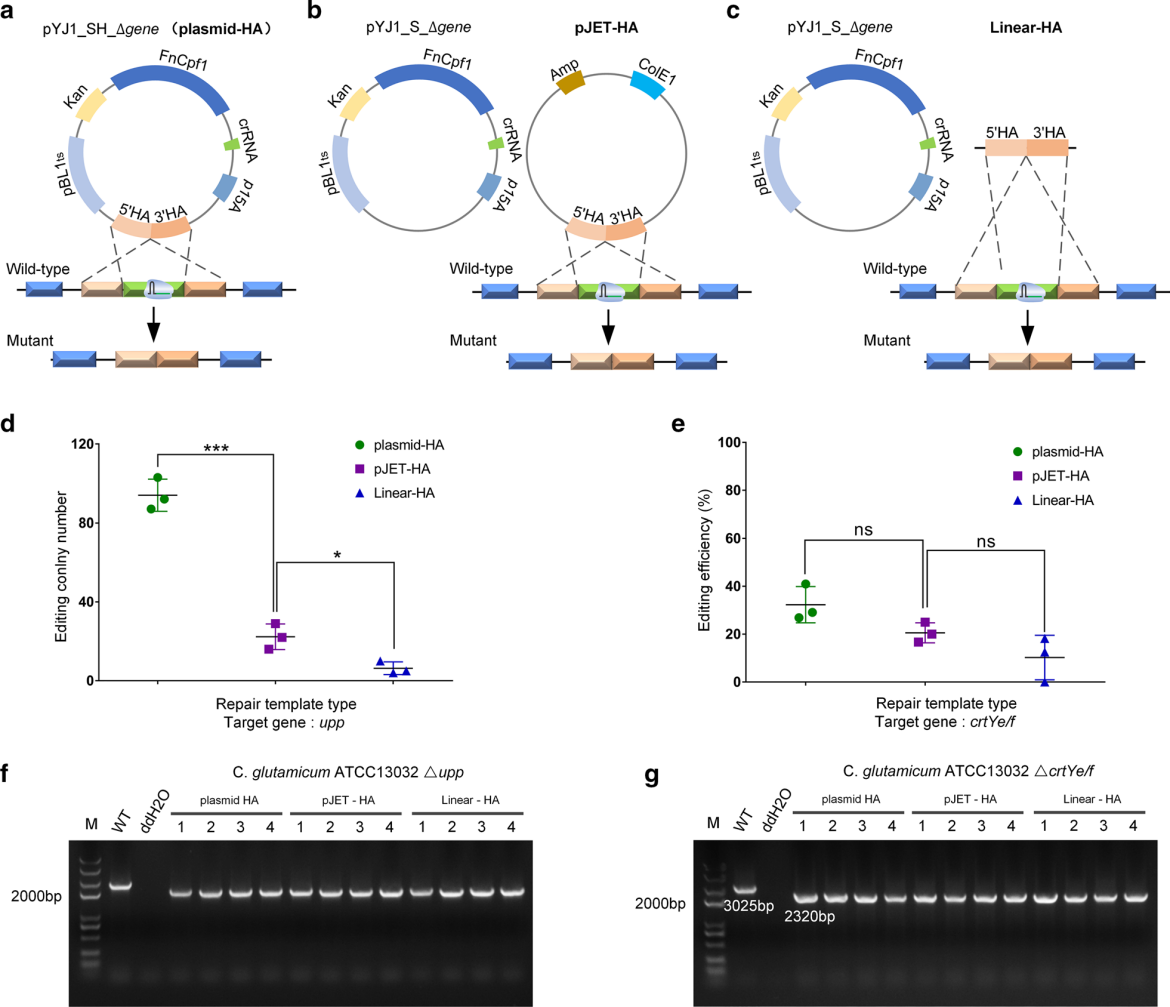
Effects of repair template type on the CRISPR-Cpf1 system editing efficiencies in *C. glutamicum*. **a**–**c** Schematic overview of CRISPR-Cpf1 dependent repair of the same DSBs using plasmid-HA (**a**), pJET-HA (**b**) or linear-HA (**c**) as templates. **d**, **e** The editing colony number when targeting the *upp* gene (**d**) and editing efficiency when targeting the *crtYe/f* gene (**e**) using different repair template types. **f** PCR validation of *upp* gene deletion using different repair template types. The F17/R17 primers bind outside of the homologous arms. The amplified fragments are 2814 bp (wild-type) and 2128 bp (edited strain). **g** PCR validation of *crtYe/f* gene deletion using different repair template types. The F10/R10 primers bind outside of the homologous arms. The amplified fragments are 3025 bp (wild-type) and 2320 bp (edited strain)

The types of DNA tested in our study were plasmid-HA, pJET-HA and linear-HA. The repair template was first ligated into the pYJ1_S_∆gene vector, which has a replicon of *C. glutamicum* (Fig. 4a) and the pJET blunt vector, which cannot replicate in *C. glutamicum* (Fig. 4b). The linearized donor was then used to determine whether it was applicable in *C. glutamicum* (Fig. 4c). The number of *upp*-edited colonies when using the linear-HA template was 6.73 ± 0.02% of the number when using the plasmid-HA template. The average number of colonies with the plasmid-HA, pJET-HA, and linear-HA templates was 94 ± 6.68, 22 ± 5.31, and 6 ± 2.62, respectively (Fig. 4d). Further, we assessed the effect of different repair templates on *crtYe/f*-editing efficiency. When using plasmid-HA, pJET-HA, and linear-HA templates, the *crtYe/f*-editing efficiencies were 32.30 ± 6.18%, 20.56 ± 3.42%, and 10.23 ± 7.59%, respectively (Fig. 4e). The same gene knockout efficiencies were obtained with in vitro linearized DNA obtained by either PCR-amplification or restriction-enzyme digestion of the donor plasmid (data not shown). These results showed that the plasmid-HA template gave the highest gene editing efficiency and the pJET-HA template gave an intermediate level of efficiency.

### Application of the CRISPR–Cpf1 method to modify multiple genes for microbial production of isobutyrate

**Fig. 5:**
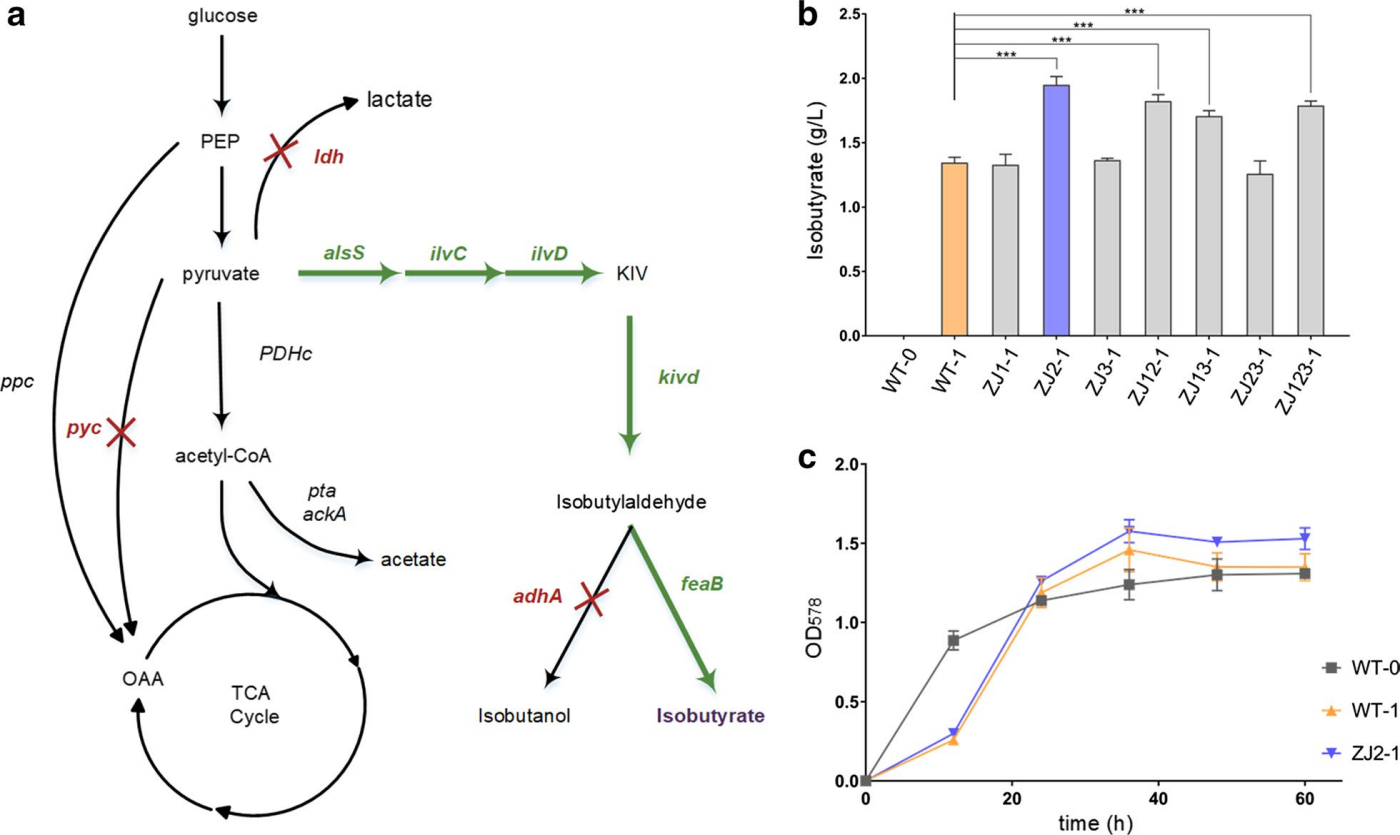
Multigene editing for isobutyrate production in *C. glutamicum* using the optimized CRISPR-Cpf1 system. **a** Schematic diagram of the central metabolic pathways and the introduced isobutyrate biosynthesis pathway in *C. glutamicum*. Gene knockout or gene expression is marked in red or green, respectively. PEP, phosphoenolpyruvate; KIV, 2-ketoisovalerate; OAA, oxaloacetate. **b** Isobutyrate production in a 60-h fermentation. ZJ0-0 represents wild-type (WT) *C. glutamicum* ATCC13032 with the empty vector, pKJ0; ZJ0-1 represents WT *C. glutamicum* ATCC13032 with pKJ1; ZJ1-1 represents WT∆*pyc* with pKJ1; ZJ2-1 represents WT∆*ldh* with pKJ1; ZJ3-1 represents WT∆*adhA* with pKJ1; ZJ12-1 represents WT∆*py*∆*ldh* with pKJ1; ZJ13-1 representsWT∆*pyc*∆*adhA* with pKJ1; ZJ23-1 represents WT∆*ldh*∆*adhA* with pKJ1; ZJ123-1 represents WT∆*pyc*∆*ldh*∆*adhA* with pKJ1. Plasmid pKJ1 contains the isobutyrate biosynthetic pathway. **c** Cell growth in a 60-h fermentation

Our CRISPR–Cpf1 mediated genome editing procedure significantly enhanced the ability to iteratively introduce well-designed genomic mutations in *C. glutamicum*. To illustrate its potential application in metabolic engineering, we used our system to modify *C. glutamicum* for the production of isobutyrate. We applied a strategy of overexpressing the 2-keto acid biosynthesis pathway (Fig. 5), which has also been engineered into *Escherichia coli*. The yield of isobutyrate in engineered *E. coli* has been reported to be approximately 4.8 g/L [38]. The 2-keto acid biosynthesis pathway includes *alsS* (*Bacillus subtilis*) and *ilvCD* (*C. glutamicum*), along with downstream genes for the subsequent decarboxylation (*kivD* from *Lactococcus lactis*) and oxidation (*feaB* from *E. coli*) of 2-ketoisovalerate to isobutyrate. The expression of these five genes was driven by the strong constitutive promoter, *Peftu*, in the plasmid, pKJ1. The empty vector, pKJ0, was used as a negative control.

The wild-type (WT) strain *C. glutamicum* with pKJ1 (WT-1) produced 1.34 ± 0.03 g/L of isobutyrate in 60 h (Fig. 5b). We then attempted to increase isobutyrate production by using the CRISPR-Cpf1 system to inactivate several enzymes that may consume precursors of the isobutyrate pathway. The genes, *pyc* (encoding pyruvate carboxylase), *ldh* (encoding lactate dehydrogenase), and *adhA* (encoding alcohol dehydrogenase) were chosen to block the competitive pathways (Fig. 5a). We constructed three single-knockout strains, named ZJ1, ZJ2, ZJ3; three double-knockout strains, named ZJ12, ZJ13, ZJ23; and one triple-knockout strain, named ZJ123 (Table 1). The ∆*pyc* and ∆*adhA* knockout strains (ZJ1 and ZJ3) and the ∆*ldh*∆*adhA* strain (ZJ23) did not show increased isobutyrate production compared with the WT strain. By contrast, isobutyrate production in the ∆*ldh* strain (ZJ2) increased to 2.01 ± 0.05 g/L (Fig. 5b), while the ∆*pyc*∆*ldh* (ZJ12), ∆*pyc*∆*adhA* (ZJ13), and ∆*pyc*∆*ldh*∆*adhA* (ZJ123) strains, containing pKJ1, produced 1.82 ± 0.04, 1.70 ± 0.03, and 1.78 ± 0.03 g/L of isobutyrate, respectively. Interestingly, the isobutyrate-producing strains also reached a higher cell density than the control strain (Fig. 5c).

**Table 1:** Strains and plasmids used in this study

Strain or plasmid	Description^a^	Reference or source
Strain		
*E. coli* Top10	General cloning host	Biomed
*C. glutamicum* ATCC 13032	Wild-type strain	ATCC
*C. glutamicum* Δ*upp*	ATCC 13032 derivative with its *upp* gene deleted	This study
*C. glutamicum* Δ*crtYe/f*	ATCC 13032 derivative with its *crtYe/f* gene deleted	This study
ZJ1	ATCC 13032 derivative with its *pyc* gene deleted	This study
ZJ2	ATCC 13032 derivative with its *ldh* gene deleted	This study
ZJ3	ATCC 13032 derivative with its *adhA* gene deleted	This study
ZJ12	ATCC 13032 derivative with its *pyc* gene and *ldh* gene deleted	This study
ZJ13	ATCC 13032 derivative with its *pyc* gene and *adhA* gene deleted	This study
ZJ23	ATCC 13032 derivative with its *ldh* gene and *adhA* gene deleted	This study
ZJ123	ATCC 13032 derivative with its *pyc* gene, *ldh* gene and *adhA* gene deleted	This study
WT-0	*C. glutamicum* ATCC 13032 + pKJ0	This study
WT-1	*C. glutamicum* ATCC 13032 + pKJ1	This study
ZJ1-1	ZJ1 + pKJ1	This study
ZJ2-1	ZJ2 + pKJ1	This study
ZJ3-1	ZJ3 + pKJ1	This study
ZJ12-1	ZJ12 + pKJ1	This study
ZJ13-1	ZJ13 + pKJ1	This study
ZJ23-1	ZJ23 + pKJ1	This study
ZJ123-1	ZJ123 + pKJ1	This study
Plasmid^b^		
pJYS3_Δ*crtYf*	pBL1^ts^ ori V _*C.glutamicum*_. Kan^R^ pSC101 ori V _*E. coli*_ PlacM-FnCpf1, Pj23119-crRNA targeting *crtYf*, 1 kb upstream and downstream homologous arms flaking the 705 bp deletion fragment inside *crtYf*	Jiang et al. [12]
pYJ1	pBL^ts^ ori V _*C.glutamicum*_ Kan^R^ p15A ori V _*E.coli*_ PlacM-*Fn*Cpf1, crRNA expression cassette	This study
pYJ1_S_Δ*upp*	pYJ1 carrying spacer sequence targeting *upp* gene	This study
pYJ1_SH_Δ*upp*	pYJ1_S_Δ*upp* carrying upstream and downstream homologous arm targeting *upp* gene	This study
pYJ1_S_Δ*crtYe/f*	pYJ1 carrying spacer sequence targeting *crtYe/f* gene	This study
pYJ1_SH_Δ*crtYe/f*	pYJ1_S_Δ*crtYe/f* carrying upstream and downstream homologous arm targeting *crtYe/f* gene	This study
pYJ1_S_Δ*pyc*	pYJ1 carrying spacer sequence targeting *pyc* gene	This study
pYJ1_SH_Δ*pyc*	pYJ1 carrying spacer sequence and homologous arm targeting *pyc* gene	This study
pYJ1_S_Δ*ldh*	pYJ1 carrying spacer sequence targeting *ldh* gene	This study
pYJ1_SH_Δ*ldh*	pYJ1 carrying spacer sequence and homologous arm targeting *ldh* gene	This study
pYJ1_S_Δ*adhA*	pYJ1 carrying spacer sequence targeting *adhA* gene	This study
pYJ1_SH_Δ*adhA*	pYJ1 carrying spacer sequence and homologous arm targeting *adhA* gene	This study
pJET_HA_Δ*upp*	pJET vector carrying upstream and downstream homologous arm targeting *upp* gene	This study
pJET_HA_Δ*crtYe/f*	pJET vector carrying upstream and downstream homologous arm targeting *crtYe/f* gene	This study
pKS167	NG2 ori; Kan^R^; Peftu::*alsS*-*ilvCD*-*kivd*-*adhA*	Smith et al. [44]
pKJ0	NG2 ori; Kan^R^; Peftu	This study
pKJ1	NG2 ori; Kan^R^; Peftu::*alsS*-*ilvCD*-*kivd*-*feaB*	This study

^a^Kan^R^ represents resistance to kanamycin

^b^crRNA expression plasmids with changed spacer sequences are not listed here. A full list of spacer sequences used in this study is shown in Additional file 1: Table S2

## Discussion

Recently, CRISPR-Cpf1 was investigated as a leading-edge tool for genome editing in many bacteria. It was reported to be suitable for *C. glutamicum*, but the efficiency of gene editing was ~ 15% when targeting a 705 bp gene [12]. Effective gene editing requires the formation of a functional complex between Cpf1, crRNA, and the target DNA strand. In this study, we systematically investigated various parameters of a *C. glutamicum* CRISPR-Cpf1 system, including the PAM sequence, the length of the spacer sequence, and the type of repair template.

FnCpf1 requires only a single crRNA, without a tracrRNA, and it recognizes thymine-rich PAM sequences. The PAM sequence is a key sequence regulating the accurate binding of crRNA to the genome. FnCpf1 may recognize the PAM sequence via shape- and base-readout mechanisms [40]. The PAM-binding channel of Cpf1 has conformational flexibility, which allows the recognition of different PAM sequences. However, the optimal PAM sequence for the manipulation of *C. glutamicum* has not yet been reported.

Therefore, we constructed a lethal reporter system and used it to examine the PAM sequence preference of CRISPR-Cpf1 in *C. glutamicum*. Using two different target genes, T was the found to be preferred at the first nucleotide position, although all nucleotides were functional at this position. T or C was the preferred at the second nucleotide position. At the third nucleotide of the PAM sequence, T is generally believed to optimal [25] and therefore, we did not test an alternative nucleotide at this position. When the last nucleotide of the PAM sequence was T, the efficiency of the crRNA was the lowest compared with other nucleotides at this position, which agreed with the results reported by Tu et al. [35]. However, crRNA had the highest efficiency of cutting target sites within the *upp* and *crtYe/f* genes when the last nucleotide of the PAM sequence was A or C. Based on these results, we concluded that the PAM sequence, 5′-NYTV-3′ (N=T/A/G/C, Y=T/C, V=A/C/G) was preferred for FnCpf1 to achieve a high targeting efficiency in the *C. glutamicum* genome. These results shed light on the PAM sequence requirements of FnCpf1 and the molecular basis of PAM recognition in *C. glutamicum*.

We further investigated the effect of different crRNA spacer sequence lengths on the efficiency of Cpf1-dependent editing. We found that FnCpf1 had the highest activity in *C. glutamicum* when a 21 bp crRNA spacer sequence was used. Structural studies have shown that only a 20 bp RNA–DNA heteroduplex is formed between the target DNA strand and a 24 bp crRNA spacer sequence [41, 42]. It was speculated that a 21 bp spacer sequence may form a complex with its target DNA strand and FnCpf1 in human cells, in which FnCpf1 has improved activity and higher fidelity [35]. This was an unexpected result which requires further investigation. When we attempted to cleave another target DNA sequence within the *upp* gene using the 5′-TTTC-3′ PAM sequence, a 20 bp crRNA spacer sequence (5′-cgtgcagcagccaacgacct) completely lost its activity, whereas other crRNAs with 17–19 bp or 21–25 bp spacer sequences showed high activity (data not shown).

If the plasmids containing the crRNA and template could be constructed independently, the speed of genome editing would be accelerated. We attempted to introduce the repair template into the pJET vector, which cannot replicate and therefore, introduces a lower metabolic burden on the host. Although the repair efficiency of pJET derivatives was not as high as that of replicable plasmid-borne templates, it was sufficient for gene editing. Finally, we assessed whether a linear repair template could be used in *C. glutamicum*. For the first time, we showed that a linear repair template could be successfully utilized to edit genomic DNA in *C. glutamicum*. However, the editing efficiency was relatively low, likely because a restriction repair system may have degraded the linear repair template prior to the genome repair process.

*C. glutamicum* is an important amino acid production strain and is highly suitable for the production of isobutyrate, which is an acid with high market demand. Here, we introduced an isobutyrate synthesis pathway into *C. glutamicum* and confirmed that the transgenic strain produced isobutyrate successfully, albeit at low rates. To further engineer a potential industrial isobutyrate production strain, we used our genome editing system to knock out the *pyc*, *ldh*, and *adhA* genes from the genome of *C. glutamicum.* Among all the host strains we constructed, the ∆*ldh* strain, ZJ2, containing pKJ1 produced the highest yield of isobutyrate. Knocking out *ldh* blocks the production of acetic acid, which increases the pyruvate pool available for isobutyrate production. However, *pyc* knockout did not significantly increase isobutyrate production in *C. glutamicum*, probably due to a reduction in the supply of oxaloacetate in the citric acid cycle, resulting in unbalanced redox levels. Similarly, *adhA* knockout did not significantly increase isobutyrate production, indicating that other enzymes with alcohol dehydrogenase activity may also be present in *C. glutamicum*. The same changes in the host genome were previously successfully introduced into *E. coli* [38, 39], but except the knockout of *ldh*, the knockout of *pyc* and *adhA* didn’t work efficiently in *C. glutamicum*. The metabolic engineering of production hosts requires multiple rounds of genome editing to optimize production. The high efficiency of the crRNA in our study helps avoid the additional workload due to false positives caused by poor crRNA design. Using linear-HA or pJET-HA can decrease the time required for plasmid construction and can potentially facilitate multiplex gene editing when constructing engineered *C. glutamicum*.

## Conclusions

In summary, we have comprehensively and quantitatively assessed the effects of PAM sequence, spacer sequence length, and repair template type on the efficiency of the CRISPR-Cpf1 system in *C. glutamicum*. Our study offers insights into FnCpf1 endonuclease function and Cpf1-based genome editing.

## Methods

### Strains and culture conditions

All bacterial strains used in this work are listed in Table 1. The Top10 *E. coli* strain was used for routine cloning procedures. *C. glutamicum* ATCC13032 was used as the parent strain for genetic modifications. All engineered strains were transformed by electroporation. All strains were kept at − 80 °C as glycerol stocks prepared in Luria-Bertani (LB) or BHIS broth containing 15% glycerol.

*E. coli* Top10 was grown aerobically in LB medium (10 g/L tryptone, 5 g/L yeast extract, and 10 g/L NaCl) at 37 °C, with shaking at 200 rpm. *C. glutamicum* ATCC 13032 strains were routinely cultivated in BHIS medium (37 g/L brain heart infusion medium and 91 g/L sorbitol) at 30 °C, with shaking at 200 rpm. Antibiotics were added at the following concentrations: 50 μg/mL kanamycin, 100 μg/mL ampicillin, and 25 μg/mL chloramphenicol for *E. coli* and 25 μg/mL kanamycin and 10 μg/mL chloramphenicol for *C. glutamicum*. Colonies with successful knockout of *upp* were selected on plates of CGXII medium (20 g/L [NH_4_]_2_SO_4_, 5 g/L urea, 1 g/L KH_2_PO_4_, 1 g/L K_2_HPO_4_, 0.25 g/L MgSO_4_·7H_2_O, 10 mg/L CaCl_2_, 10 mg/L FeSO_4_·7H_2_O, 10 mg/L MnSO_4_·H_2_O, 1 mg/L ZnSO_4_·7H_2_O, 0.2 mg/L CuSO_4_·5H_2_O, 0.02 mg/L NiCl_2_·6H_2_O, 0.2 mg/L biotin, 42 g/L 3-morpholinopropanesulfonic acid [MOPS], and 40 g/L glucose), containing 100 μM 5-FU (from a 100 mM stock in dimethyl sulfoxide). *C. glutamicum* fermentations were also performed in CGXII medium.

### Plasmid constructions

All plasmids used in this study are listed in Table 1. The sequences of the primers, crRNAs, and oligonucleotides are listed in Additional file 1: Tables S1 and S2. Plasmids and genomic DNA were extracted using the TIANamp Mini Plasmid Kit (TIANGEN BIOTECH, Beijing, China) and TIANamp Bacteria DNA Kit (TIANGEN BIOTECH), according to the manufacturer’s instructions. PCR was performed using TransStart FastPfu Fly DNA Polymerase (TransGen Biotech, Beijing, China). DNA was extracted from agarose gels using the HiPure Gel Pure DNA Micro Kit (Magen, Guangzhou, China). Cloning was performed using commercially available restriction endonucleases, T4 DNA ligase, the CloneJET PCR Cloning Kit (Thermo Scientific, Waltham, MA, USA), and the Gibson assembly method [43].

The plasmid, pYJ1, was constructed via Gibson assembly using the following three DNA fragments: a Cpf1 expression cassette with a temperature-sensitive replicon; pBL^ts^ with a kanamycin resistance gene fragment, originally constructed by Jiang et al. [12]; a *E. coli* replicon p15A and a crRNA expression cassette. The plasmid, pJYS3_∆*crtYf* [12], was used as a template to amplify the fragment using F1/R1 primers and an ApaI restriction enzyme cutting site was added to the primers to insert homologous arms. The crRNA expression cassette consisted of the constitutive promoter, pJ23119; a 19 bp direct repeat sequence of the crRNA; a 25 bp sequence with an AarI restriction enzyme site to insert the spacer sequence, and an rrnB terminator. The nucleotide sequences of the crRNA expression cassettes used in this study are listed in Additional file 1: Table S3. To generate pYJ1_S_∆gene, pYJ1 was linearized with AarI and a pair of primers (such as F4/R4) was annealed to form a dsDNA fragment with cohesive ends, which was then ligated to pYJ1 using T4 DNA ligase. To generate pYJ1_SH_∆gene, pYJ1_S_∆gene was linearized with *Apa*I. The *C. glutamicum* ATCC13032 genome was used as a template to amplify 500 bp upstream and downstream homologous arms using corresponding primers (such as F5/R5 and F6/R6, respectively). The *Apa*I restriction enzyme site was introduced by the primers and the two fragments were assembly via another round of PCR. The purified PCR product was then cut using *Apa*I and inserted into the linearized pYJ1_S_∆gene plasmid. To construct pJET_HA_∆gene, homologous arms were amplified using the primers pairs like F15/R15 and the PCR products were ligated to the vector, pJET1.2 (CloneJET PCR Cloning Kit, Thermo Fisher Scientific). To construct the plasmid, pKJ0, the backbone of pKS167 was amplified using the primer pair, F33/R33 [44] and the PCR product was used directly to transform competent *E. coli* cells. The plasmid was verified by PCR and sequencing. To construct the plasmid, pKJ1, the backbone of pKS167 was amplified using the primer pair, F35/R35. The *feaB* gene was amplified from the *E. coli* genome using the primer pair, F36/R36. The two fragments were then assembled using the Gibson assembly method.

### Iterative genome editing procedure

Electrocompetent cells were generated as described previously [45]. In the plasmid-borne template system, 500 ng of pYJ1_S_∆gene or pYJ1_SH_∆gene was used for each electroporation. For the linear-HA and pJET-HA templates, 500 ng of pYJ1_S_∆gene and 1 μg of the repair template were mixed together to co-transform competent cells. Electroporation was performed using a Gene Pulser system (Bio-Rad, Hercules, CA, USA) set to 25 μF, 1.25 kV/cm, and 200Ω. Following exposure to a single electric pulse, the cell suspension was immediately transferred into 1 mL of pre-warmed (46 °C) BHIS medium and incubated for 6 min at 46 °C without shaking. Subsequently, *C. glutamicum* cells were incubated with shaking at 200 rpm for 2 h at 30 °C. Finally, cells were spread on selective BHIS agar plates containing 25 μg/mL kanamycin. Transformants were confirmed by PCR amplification.

For plasmid curing, colonies were seeded into BHIS without antibiotics and cultivated for 6–8 h or overnight at 37 °C. To save time, the colonies were then simultaneously inoculated for plasmid curing to allow iterative engineering. After plasmid curing, the cultures were streaked and the colonies were tested for kanamycin sensitivity. The edited colony was then grown to mid-log phase to prepare electrocompetent cells for the next round of editing.

To verify these editing events by Sanger sequencing, colonies were picked and the target region was amplified by PCR. The primers used for PCR amplification and sequencing are listed in Additional file 1: Table S1.

### Determining crRNA targeting efficiency by measuring Cpf1-induced lethality

Plasmids carrying the crRNA expression cassette were introduced into competent cells (500 ng plasmid/100 μL of competent cells). After transformation, cells were incubated in BHIS medium for 1 h at 30 °C for recovery. The culture was then streaked on BHIS agar plates containing kanamycin. The colonies were counted after 36–48 h of cultivation and the survival rates for each crRNA were calculated by counting the colonies that formed. To minimize the impact of differences in electroporation efficiency during the preparation of competent cells, the survival rates were further normalized by determining the colony number after transformation with a negative control crRNA plasmid. All transformations were performed in triplicate. The survival rate was calculated according to the following formula:

$${\text{Survival rate }} = \, \left( {\left[ {\text{read count}} \right]_{\text{selective}} /{\text{average}}\left[ {\text{read count}} \right]_{\text{control}} } \right) \times 100\% .$$

### Shake-flask fermentation

Five milliliters of BHIS medium was inoculated with a single colony of the desired *C. glutamicum* strain from a fresh BHIS agar plate and the culture was incubated on a rotary shaker at 30 °C overnight. For isobutyrate production, 200 μL of the overnight culture was transferred into a 250-mL anaerobic shake flask with 20 mL of CGXII medium, containing 40 g/L glucose. Fermentation was performed at 30 °C, with shaking at 200 rpm. Samples were drawn every 12 h to determine metabolite concentrations and perform OD_578_ measurements. All experiments were performed in at least three biological replicates.

### Metabolic analysis

Organic acids in the culture supernatants were quantified using an Agilent 1260 Infinity HPLC system, equipped with an RID detector and an Aminex HPX-87H cation exchange column (BioRad). The samples were first filtered through a 0.22-μm filter, then loaded onto the column operated at 60 °C, eluted with 5 mM H_2_SO_4_ at a flow rate of 0.5 mL/min, and detected via refractive index. The data are presented as mean values, with error bars indicating standard deviation.

## Additional file

**Additional file 1.** Additional Figures S1, S2 and Tables S1–S3.

## Abbreviations

CRISPR: clustered regularly interspaced short palindromic repeats
FnCpf1: Cpf1 derived from *F. novicida* (NC_008601))
crRNA: CRISPR RNA
tracrRNA: trans-activating CRISPR RNA
PAM: protospacer adjacent motif
DSBs: double-strand breaks
dsDNA: double strand DNA
HR: homologous recombination
OD_578_: optical density at 578 nm
HPLC: high-pressure liquid chromatography detector
RID: refractive index detector
Kan: kanamycin
PCR: polymerase chain reaction
5-FU: 5-fluorouracil
PEP: phosphoenolpyruvate
KIV: 2-ketoisovalerate
OAA: oxaloacetate
