# Supplementary material for: Optimizing a CRISPR-Cpf1-based genome engineering system for Corynebacterium glutamicum
Source: Microb Cell Fact. 2019 Mar 25;18:60. doi: 10.1186/s12934-019-1109-x (PMC6432761; doi:10.1186/s12934-019-1109-x)
Supplement: Supplementary file 1 — Additional file 1. Additional Figures S1, S2 and Tables S1–S3. [file 12934_2019_1109_MOESM1_ESM.docx]

**Additional file 1**

**Optimizing a CRISPR-Cpf1-based genome engineering system for *Corynebacterium glutamicum***

Jiao Zhang^1^, Fayu Yang^1^, Yunpeng Yang^1^, Yu Jiang^3^, Yi-Xin Huo^1,2^ *

^1^ Key Laboratory of Molecular Medicine and Biotherapy, Department of Biology, School of Life Sciences, Beijing Institute of Technology, No. 5 South Zhongguancun Street, 100081 Beijing, China

^2^ UCLA Institute of Advancement (Suzhou), 10 Yueliangwan Road, Suzhou Industrial Park, 215123 Suzhou, China

^3^ Key Laboratory of Synthetic Biology, Institute of Plant Physiology and Ecology, Shanghai Institutes for Biological Sciences, Chinese Academy of Sciences, 200032 Shanghai, China.

*Correspondence: huoyixin@bit.edu.cn

**Table of Contents**

Figure S1

Figure S2

Table S1

Table S2

Table S3


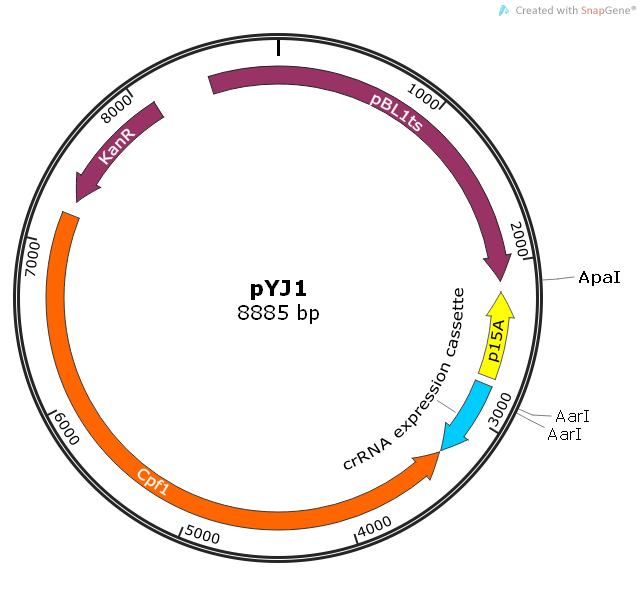


**Figure S1.** Schematic representation of Cpf1 and gRNA expression plasmids used in this study.


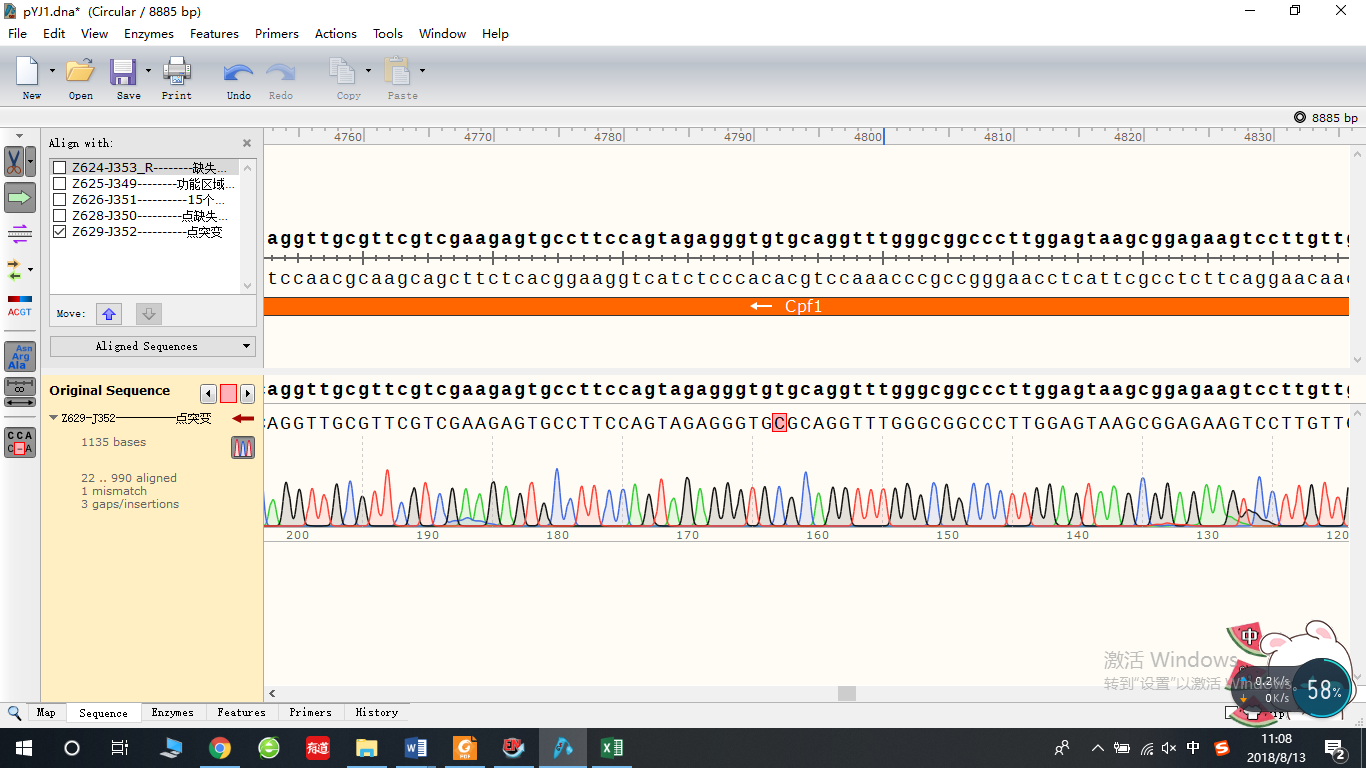

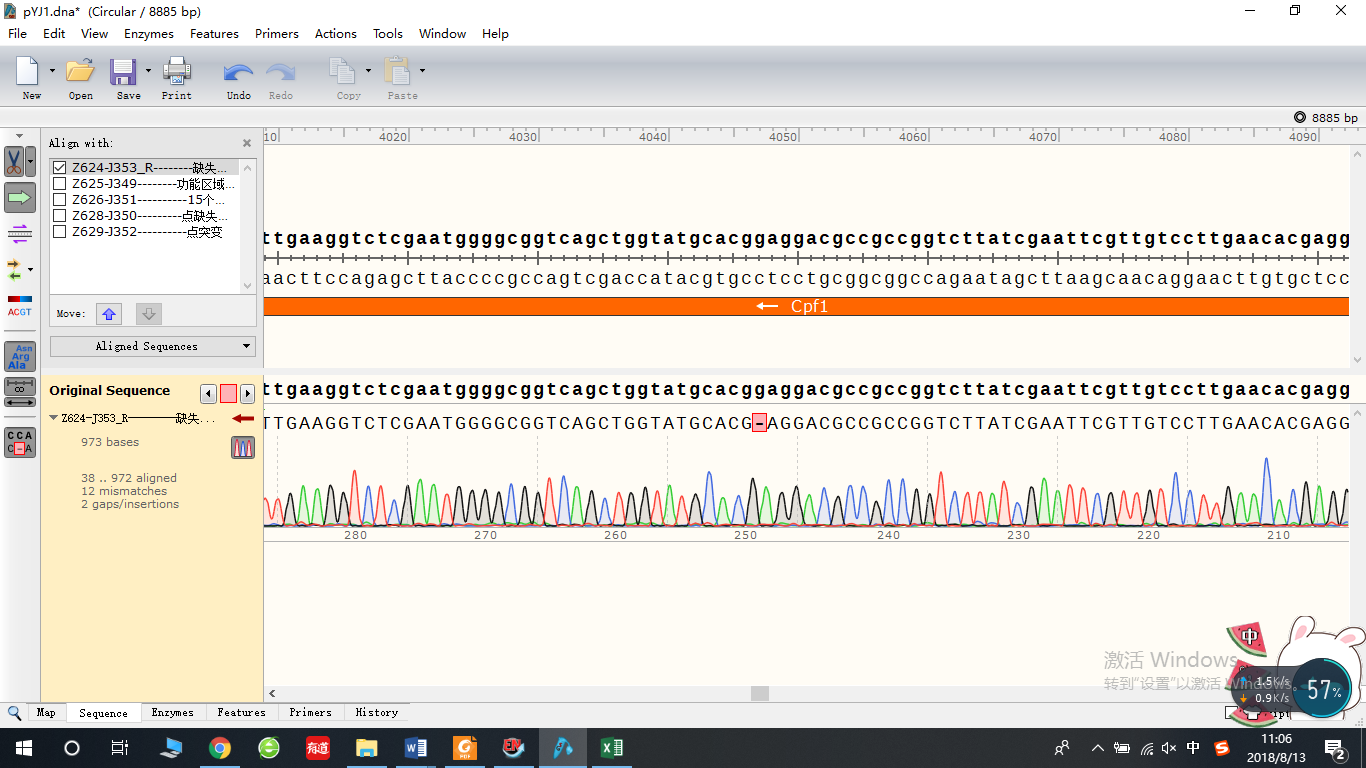


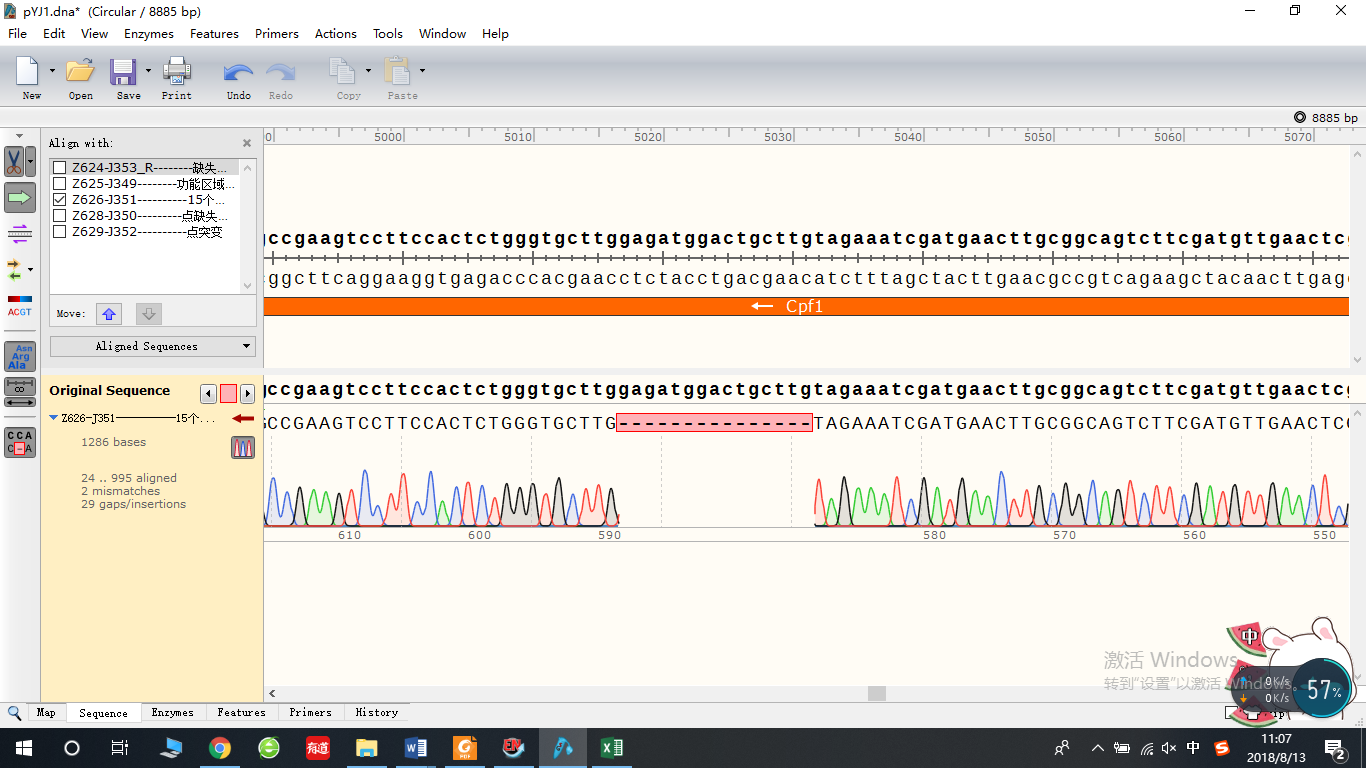


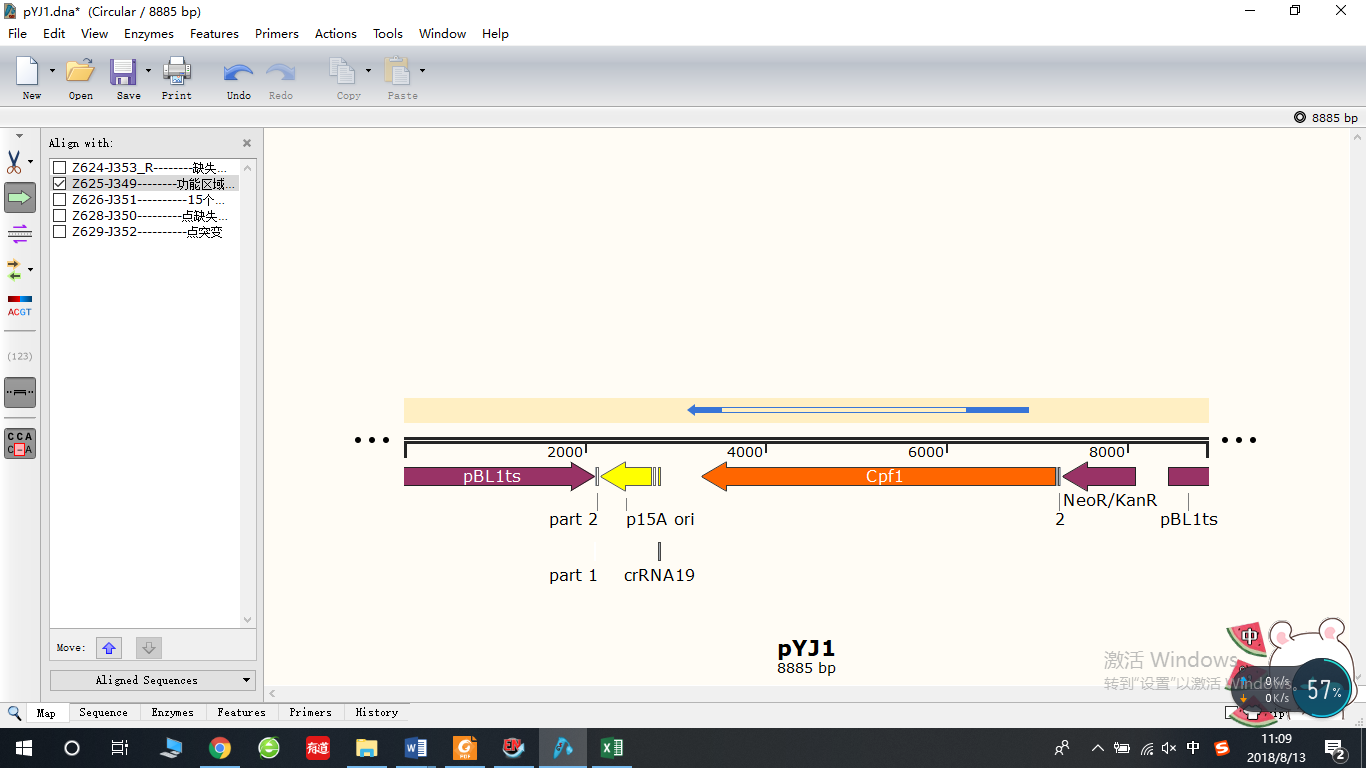


**Figure S2.** Sequencing results of diferent patterns of Cpf1 mutation. Nonsense mutation, a G4792G mutation occurred, generating another amino acid (Val to His) in Cpf1 gene. Base deletion, 1 bp, 15 bp, and 2701 bp deleted from Cpf1 gene which deactivated Cpf1.

**Table S1**

Primers used for plasmids construction

| **Plasmid** | **Primer Name** | **Primer sequence (5`-3`)** |
| --- | --- | --- |
| PYJ1 | F1 | tttgcgtttctacaaactctgcctgcaggtcgacttagtt |
|  | R1 | cttccaggggcccgccaacaacaagacccatcat |
|  | F2 | gttggcgggcccctggaagatgccaggaagat |
|  | R2 | aggactgagctagctgtcaaacgtgagttttcgttccact |
|  | crRNA-1 | ttgacagctagctcagtcctaggtataatggatccgaatttctactgttgtagatatgcg |
|  | crRNA-2 | cgccaaaacagccaaatgcaggtgtttcacctgcgcatatctacaacagtagaaattc |
|  | crRNA-3 | acctgcatttggctgttttggcggatgagagaagattttcagcctgatacagattaaatc |
|  | crRNA-4 | gcaaattctgttttatcagaccgcttctgcgttctgatttaatctgtatcaggctgaaaa |
|  | crRNA-5 | agcggtctgataaaacagaatttgcctggcggcagtagcgcggtggtcccacctg |
|  | crRNA-6 | gcgctacggcgtttcacttctgagttcggcatggggtcaggtgggaccaccgcgct |
|  | crRNA-7 | gaagtgaaacgccgtagcgccgatggtagtgtggggtctccccatgcgagagtagg |
|  | crRNA-8 | ttcgactgagcctttcgttttatttgatgcctggcagttccctactctcgcatggggaga |
|  | crRNA-9 | aataaaacgaaaggctcagtcgaaagactgggcctttcgttttatctgttgtttgtcggt |
|  | crRNA-10 | ccggcggatttgtcctactcaggagagcgttcaccgacaaacaacagataaaacga |
|  | crRNA-11 | gagtaggacaaatccgccgggagcggatttgaacgttgcgaagcaacggcccgga |
|  | crRNA-12 | cctggcagtttatggcgggcgtcctgcccgccaccctccgggccgttgcttcgca |
|  | crRNA-13 | cccgccataaactgccaggcatcaaattaagcagaaggccatcctgacggatggc |
|  | crRNA-14 | agagtttgtagaaacgcaaaaaggccatccgtcaggatggcc |
|  | F3 | acatagacgctgatgtacgt |
|  | R3 | tgtcctactcaggagagcgt |
| pYJ1_S_∆*crtYe/f* | F4 | agatcaggcaaccatagggcaggaa |
|  | R4 | agccttcctgccctatggttgcctg |
| pYJ1_SH_∆*crtYe/f* | F5 | tgggcccgtgggtggctaggcaagttac |
|  | R5 | cattgggtgcgatccaccaccacgcaatct |
|  | F6 | gtggtggatcgcacccaatgagaactagga |
|  | R6 | tgggccccctatccagcagtcttcctgtc |
|  | F7 | acatagacgctgatgtacgt |
| pJET_HA_∆*crtYe/f* | F8 | tgggcccgtgggtggctaggcaagttac |
|  | R8 | tgggccccctatccagcagtcttcctgtc |
|  | F9 | tgaacaccatatccatccggcgta |
|  | R9 | taatcggtgcaacgatcact |
| screen for knockout *crtYe/f* | F10 | gttgctcctgcgagtttgc |
|  | R10 | gcttcaccgggatgtactgac |
| pYJ1_S_∆*upp* | F11 | agatcgtgcagcagccaacgacctc |
|  | R11 | agccgaggtcgttggctgctgcacg |
| pYJ1_SH_∆*upp* | F12 | tgggcccgaagttcgtcgtaggtgacttg |
|  | R12 | gcccactacaaatcattgccgcccagaa |
|  | F13 | ggcaatgatttgtagtgggcggtgagtat |
|  | R13 | tgggcccctcgtttggttccggagtatt |
|  | F14 | acatagacgctgatgtacgt |
|  | R14 | gcgttgcccttgttgttt |
| pJET_HA_∆*upp* | F15 | tgggcccgaagttcgtcgtaggtgacttg |
|  | R15 | tgggcccctcgtttggttccggagtatt |
|  | F16 | tgaacaccatatccatccggcgta |
|  | R16 | gcgttgcccttgttgttt |
| screen for knockout *upp* | F17 | catgcttgtcgtcggattcta |
|  | R17 | tcgttgtgctgtgggtattc |
| pYJ1_S_∆*pyc* | F18 | agatcccgggatacggcttcctgtc |
|  | R18 | agccgacaggaagccgtatcccggg |
| pYJ1_SH_∆*pyc* | F19 | ctgggccccgttgatgactgttggttccat |
|  | R19 | aactcacccatctcctagagtaattattcctttcaacaagag |
|  | F20 | aattactctaggagatgggtgagttggattt |
|  | R20 | atgggcccttgtgtcattgccttgtcatatc |
|  | F21 | acatagacgctgatgtacgt |
|  | R21 | gtgaaaacccaggagtgcac |
| screen for knockout *pyc* | F22 | ctccccacccaccttatcgt |
|  | R22 | agcccgatcgcagctctcat |
| pYJ1_S_∆*ldh* | F23 | agattagttcgacaacgcggcggatgc |
|  | R23 | agccgcatccgccgcgttgtcgaacta |
| pYJ1_SH_∆*ldh* | F24 | ctgggccctgacttcaatcggcagagc |
|  | R24 | ggagttgcatacaaatcaccgaccacgagatg |
|  | F25 | tcggtgatttgtatgcaactccaacatctcct |
|  | R25 | atgggccctgggaacaccatgcgattaa |
|  | F26 | acatagacgctgatgtacgt |
|  | R26 | gtctccccaacccattacgt |
| screen for knockout *ldh* | F27 | gatggatcgtaggtgagttcttc |
|  | R27 | cgtgtcagaaagcctggtta |
| pYJ1_S_∆*adhA* | F28 | agatccgtgaatgcgcgtaatgaag |
|  | R28 | agcccttcattacgcgcattcacgg |
| pYJ1_SH_∆*adhA* | F29 | ctgggcccacgggaatcttcaggatcaa |
|  | R29 | aagcgagaaggagggattgtgttgaaactgctctg |
|  | F30 | ttcaacacaatccctccttctcgcttggattactt |
|  | R30 | atgggcccctcgggtttatctctcctgttc |
|  | F31 | acatagacgctgatgtacgt |
|  | R31 | taagccggcctcagagctgt |
| screen for knockout *adhA* | F32 | aagctggcatcctccatatc |
|  | R32 | ctcgggtttatctctcctgttc |
| pKJ0 | F33 | cactcgacgggcccactagtggctgttttggcggatgaga |
|  | R33 | aaacagccactagtgggcccgtcgagtgtatgtcctcctg |
|  | F34 | tggtcttcgattcctacgct |
|  | R34 | ggcagggcagcttatatgct |
| pKJ1 | F35 | tgaagtccaggagggctgcaatgacagagccgcatgtagc |
|  | R35 | cagtttaataccgtacacacaccgacttag |
|  | F36 | cggtgtgtgtacggtattaaactggctgttttggcggatgagag |
|  | R36 | attgcagccctcctggacttcactagtttatgat |
|  | F37 | gcgggtcgaatggaccagcc |
|  | R37 | tgtcctactcaggagagcgt |

**Table S2**

Primers with spacer sequence targeting different sites of gene

| **Primer Name** | **Primer sequence (5`-3`)** | **PAM sequence** | **The length of spacer sequence (bp)** |
| --- | --- | --- | --- |
| F(△crtYf-1) | agatCCTGCGTTTAAACATATTTCC | TTTT | 21 |
| R(△crtYf-1) | agccGGAAATATGTTTAAACGCAGG |  |  |
| F(△crtYf-2) | agatCTGCGTTTAAACATATTTCCA | TTTC | 21 |
| R(△crtYf-2) | agccTGGAAATATGTTTAAACGCAG |  |  |
| F(△crtYf-3) | agatCCAGGCAACCATAGGGCAGGA | ATTT | 21 |
| R(△crtYf-3) | agccTCCTGCCCTATGGTTGCCTGG |  |  |
| F(△crtYf-4) | agatCAGGCAACCATAGGGCAGGAA | TTTC | 21 |
| R(△crtYf-4) | agccTTCCTGCCCTATGGTTGCCTG |  |  |
| F(△crtYf-5) | agatGGCTCGGTTTAATCCCCCTAG | ATTT | 21 |
| R(△crtYf-5) | agccCTAGGGGGATTAAACCGAGCC |  |  |
| F(△crtYf-6) | agatGCTCGGTTTAATCCCCCTAGA | TTTG | 21 |
| R(△crtYf-6) | agccTCTAGGGGGATTAAACCGAGC |  |  |
| F(△crtYf-7) | agatGGATATGGCGATACCCAGCAC | CTTT | 21 |
| R(△crtYf-7) | agccGTGCTGGGTATCGCCATATCC |  |  |
| F(△crtYf-8) | agatGATATGGCGATACCCAGCACC | TTTG | 21 |
| R(△crtYf-8) | agccGGTGCTGGGTATCGCCATATC |  |  |
| F(△crtYf-9) | agatAGCAATAGCCATGGTCCTATT | TTTT | 21 |
| R(△crtYf-9) | agccAATAGGACCATGGCTATTGCT |  |  |
| F(△crtYf-10) | agatGCAATAGCCATGGTCCTATTT | TTTA | 21 |
| R(△crtYf-10) | agccAAATAGGACCATGGCTATTGC |  |  |
| F(△crtYf-11) | agatAGGCAGTGGTGCTTTAAGCCA | TTTT | 21 |
| R(△crtYf-11) | agccTGGCTTAAAGCACCACTGCCT |  |  |
| F(△crtYf-12) | agatGGCAGTGGTGCTTTAAGCCAT | TTTA | 21 |
| R(△crtYf-12) | agccATGGCTTAAAGCACCACTGCC |  |  |
| F(△upp-1) | agatCCGTGCAGCAGCCAACGACCT | CTTT | 21 |
| R(△upp-1) | agccAGGTCGTTGGCTGCTGCACGG |  |  |
| F(△upp-2) | agatCGTGCAGCAGCCAACGACCTC | TTTC | 21 |
| R(△upp-2) | agccGAGGTCGTTGGCTGCTGCACG |  |  |
| F(△upp-3) | agatCCTCATCGCGGGCAAGGCCAA | GTTT | 21 |
| R(△upp-3) | agccTTGGCCTTGCCCGCGATGAGG |  |  |
| F(△upp-4) | agatCTCATCGCGGGCAAGGCCAAT | TTTC | 21 |
| R(△upp-4) | agccATTGGCCTTGCCCGCGATGAG |  |  |
| F(△upp-5) | agatGGTGTCGAAGTGTTCGACTTC | TTTT | 21 |
| R(△upp-5) | agccGAAGTCGAACACTTCGACACC |  |  |
| F(△upp-6) | agatGTGTCGAAGTGTTCGACTTCC | TTTG | 21 |
| R(△upp-6) | agccGGAAGTCGAACACTTCGACAC |  |  |
| F(△upp-7) | agatGGTTACCGCCACCATCGACCC | GTTT | 21 |
| R(△upp-7) | agccGGGTCGATGGTGGCGGTAACC |  |  |
| F(△upp-8) | agatGTTACCGCCACCATCGACCCA | TTTG | 21 |
| R(△upp-8) | agccTGGGTCGATGGTGGCGGTAAC |  |  |
| F(△upp-9) | agatAGGCGGCTAGCAACGAGTGGG | GGTT | 21 |
| R(△upp-9) | agccCCCACTCGTTGCTAGCCGCCT |  |  |
| F(△upp-10) | agatGGCGGCTAGCAACGAGTGGGT | GTTA | 21 |
| R(△upp-10) | agccACCCACTCGTTGCTAGCCGCC |  |  |
| F(△upp-11) | agatAGATCCTGTGGCAGCGCCTCA | GCTT | 21 |
| R(△upp-11) | agccTGAGGCGCTGCCACAGGATCT |  |  |
| F(△upp-12) | agatGATCCTGTGGCAGCGCCTCAA | CTTA | 21 |
| R(△upp-12) | agccTTGAGGCGCTGCCACAGGATC |  |  |
| F(△crtYf-13) | agatAAAATATAAGTCATGGTTCAA | TATA | 21 |
| R(△crtYf-13) | agccTTGAACCATGACTTATATTTT |  |  |
| F(△crtYf-14) | agatAGTCATGGTTCAACCTCGGGA | TATA | 21 |
| R(△crtYf-14) | agccTCCCGAGGTTGAACCATGACT |  |  |
| F(△crtYf-15) | agatTCCCATAAAAGAAATCCAATA | TATA | 21 |
| R(△crtYf-15) | agccTATTGGATTTCTTTTATGGGA |  |  |
| F(△crtYf-16) | agatGCAATAGCCATGGTCCTATTT | TTTA | 21 |
| R(△crtYf-16) | agccAAATAGGACCATGGCTATTGC |  |  |
| F(△crtYf-17) | agatGGCAGTGGTGCTTTAAGCCAT | TTTA | 21 |
| R(△crtYf-17) | agccATGGCTTAAAGCACCACTGCC |  |  |
| F(△crtYf-18) | agatAGCCATAATGCTGCTGCCGAG | TTTA | 21 |
| R(△crtYf-18) | agccCTCGGCAGCAGCATTATGGCT |  |  |
| F(△crtYf-19) | agatTCCGCTCTTCGCAGTACTTCT | TCTA | 21 |
| R(△crtYf-19) | agccAGAAGTACTGCGAAGAGCGGA |  |  |
| F(△crtYf-20) | agatGGGGGATTAAACCGAGCCAAA | TCTA | 21 |
| R(△crtYf-20) | agccTTTGGCTCGGTTTAATCCCCC |  |  |
| F(△crtYf-21) | agatTTTGGATATGGCGATACCCAG | TCTC | 21 |
| R(△crtYf-21) | agccCTGGGTATCGCCATATCCAAA |  |  |
| F(△crtYf-22) | agatGCAGAGGAAGAATAAGAAAAA | TGTA | 21 |
| R(△crtYf-22) | agccTTTTTCTTATTCTTCCTCTGC |  |  |
| F(△crtYf-23) | agatTTAAAGCTGCAGTCTGGAACA | TGTC | 21 |
| R(△crtYf-23) | agccTGTTCCAGACTGCAGCTTTAA |  |  |
| F(△crtYf-24) | agatAGTGCCCTTACCCTATGTTCC | TGTC | 21 |
| R(△crtYf-24) | agccGGAACATAGGGTAAGGGCACT |  |  |
| F(△upp-13) | agatCGCCTCCCTTGCCACCTATGC | TATC | 21 |
| R(△upp-13) | agccGCATAGGTGGCAAGGGAGGCG |  |  |
| F(△upp-14) | agatCACCAGGCACTATCCGCCTCC | TATC | 21 |
| R(△upp-14) | agccGGAGGCGGATAGTGCCTGGTG |  |  |
| F(△upp-15) | agatAAACTGCGCGCCAGAATCAGT | TATC | 21 |
| R(△upp-15) | agccACTGATTCTGGCGCGCAGTTT |  |  |
| F(△upp-16) | agatACATGTGAAGCTATGGACATC | TTTA | 21 |
| R(△upp-16) | agccGATGTCCATAGCTTCACATGT |  |  |
| F(△upp-17) | agatGGCGGCTAGCAACGAGTGGGT | GTTA | 21 |
| R(△upp-17) | agccACCCACTCGTTGCTAGCCGCC |  |  |
| F(△upp-18) | agatGATCCTGTGGCAGCGCCTCAA | CTTA | 21 |
| R(△upp-18) | agccTTGAGGCGCTGCCACAGGATC |  |  |
| F(△upp-19) | agatAGCAACCAGCCTGTATTCCTT | TCTA | 21 |
| R(△upp-19) | agccAAGGAATACAGGCTGGTTGCT |  |  |
| F(△upp-20) | agatGACGAAAACGCCTACATCGTG | TCTA | 21 |
| R(△upp-20) | agccCACGATGTAGGCGTTTTCGTC |  |  |
| F(△upp-21) | agatCGGTCCGCGAAACATCGACCT | TCTA | 21 |
| R(△upp-21) | agccAGGTCGATGTTTCGCGGACCG |  |  |
| F(△upp-22) | agatTTCCTTGTCGATCCCATGCTG | TGTA | 21 |
| R(△upp-22) | agccCAGCATGGGATCGACAAGGAA |  |  |
| F(△upp-23) | agatGGCGTTTTCGTCTAGACCTGG | TGTA | 21 |
| R(△upp-23) | agccCCAGGTCTAGACGAAAACGCC |  |  |
| F(△upp-24) | agatGTGGGCGGTGAGTATTCTTAT | TGTA | 21 |
| R(△upp-24) | agccATAAGAATACTCACCGCCCAC |  |  |
| F(△crtYf-25) | agatAACCGAGCCAAATGCCAAGGT | ATTA | 21 |
| R(△crtYf-25) | agccACCTTGGCATTTGGCTCGGTT |  |  |
| F(△crtYf-26) | agatACCTTGCACCCCATATGCCCA | ATTA | 21 |
| R(△crtYf-26) | agccTGGGCATATGGGGTGCAAGGT |  |  |
| F(△crtYf-27) | agatCTGGCACTTTTTATCGCGGAG | ATTA | 21 |
| R(△crtYf-27) | agccCTCCGCGATAAAAAGTGCCAG |  |  |
| F(△crtYf-28) | agatGCAATAGCCATGGTCCTATTT | TTTA | 21 |
| R(△crtYf-28) | agccAAATAGGACCATGGCTATTGC |  |  |
| F(△crtYf-29) | agatGGCAGTGGTGCTTTAAGCCAT | TTTA | 21 |
| R(△crtYf-29) | agccATGGCTTAAAGCACCACTGCC |  |  |
| F(△crtYf-30) | agatAGCCATAATGCTGCTGCCGAG | TTTA | 21 |
| R(△crtYf-30) | agccCTCGGCAGCAGCATTATGGCT |  |  |
| F(△crtYf-31) | agatCCCTATGTTCCCTAACTATCA | CTTA | 21 |
| R(△crtYf-31) | agccTGATAGTTAGGGAACATAGGG |  |  |
| F(△crtYf-32) | agatTATTTTTATAAGCATTCCTTT | CTTA | 21 |
| R(△crtYf-32) | agccAAAGGAATGCTTATAAAAATA |  |  |
| F(△crtYf-33) | agatAAGCACCACTGCCTAAAAAAC | CTTA | 21 |
| R(△crtYf-33) | agccGTTTTTTAGGCAGTGGTGCTT |  |  |
| F(△crtYf-34) | agatGGGAACATAGGGTAAGGGCAC | GTTA | 21 |
| R(△crtYf-34) | agccGTGCCCTTACCCTATGTTCCC |  |  |
| F(△crtYf-35) | agatATACCGGACATAAACGCTGAG | GTTA | 21 |
| R(△crtYf-35) | agccCTCAGCGTTTATGTCCGGTAT |  |  |
| F(△crtYf-36) | agatCGAGCAATTCTTTCGGTAGGT | GTTA | 21 |
| R(△crtYf-36) | agccACCTACCGAAAGAATTGCTCG |  |  |
| F(△upp-25) | agatATTTGCCCCACGCCAAAGAGC | ATTA | 21 |
| R(△upp-25) | agccGCTCTTTGGCGTGGGGCAAAT |  |  |
| F(△upp-26) | agatCGGAAAATTCTTGCCCACTGT | ATTA | 21 |
| R(△upp-26) | agccACAGTGGGCAAGAATTTTCCG |  |  |
| F(△upp-27) | agatGAAATGGTGTTTCGGGACACT | ATTA | 21 |
| R(△upp-27) | agccAGTGTCCCGAAACACCATTTC |  |  |
| F(△upp-28) | agatACATGTGAAGCTATGGACATC | TTTA | 21 |
| R(△upp-28) | agccGATGTCCATAGCTTCACATGT |  |  |
| F(△upp-29) | agatAAAGTTTCTAAGGGCATTACG | TTTA | 21 |
| R(△upp-29) | agccCGTAATGCCCTTAGAAACTTT |  |  |
| F(△upp-30) | agatAAGGTCGATGTTTCGCGGACC | TTTA | 21 |
| R(△upp-30) | agccGGTCCGCGAAACATCGACCTT |  |  |
| F(△upp-31) | agatGATCCTGTGGCAGCGCCTCAA | CTTA | 21 |
| R(△upp-31) | agccTTGAGGCGCTGCCACAGGATC |  |  |
| F(△upp-32) | agatGAAACTTTTAAAGGTCGATGT | CTTA | 21 |
| R(△upp-32) | agccACATCGACCTTTAAAAGTTTC |  |  |
| F(△upp-33) | agatTGCATCGGCAATGTTGTCTAT | CTTA | 21 |
| R(△upp-33) | agccATAGACAACATTGCCGATGCA |  |  |
| F(△upp-34) | agatGGCGGCTAGCAACGAGTGGGT | GTTA | 21 |
| R(△upp-34) | agccACCCACTCGTTGCTAGCCGCC |  |  |
| F(△upp-35) | agatAATCATTGCCGCCCAGAAGAA | GTTA | 21 |
| R(△upp-35) | agccTTCTTCTGGGCGGCAATGATT |  |  |
| F(△upp-36) | agatCCGCCACCATCGACCCAGGTC | GTTA | 21 |
| R(△upp-36) | agccGACCTGGGTCGATGGTGGCGG |  |  |
| F(△crtYf-37) | agatCCCTATGTTCCCTAACT | CTTA | 17 |
| R(△crtYf-37) | agccAGTTAGGGAACATAGGG |  |  |
| F(△crtYf-38) | agatCCCTATGTTCCCTAACTA | CTTA | 18 |
| R(△crtYf-38) | agccTAGTTAGGGAACATAGGG |  |  |
| F(△crtYf-39) | agatCCCTATGTTCCCTAACTAT | CTTA | 19 |
| R(△crtYf-39) | agccATAGTTAGGGAACATAGGG |  |  |
| F(△crtYf-40) | agatCCCTATGTTCCCTAACTATC | CTTA | 20 |
| R(△crtYf-40) | agccGATAGTTAGGGAACATAGGG |  |  |
| F(△crtYf-41) | agatCCCTATGTTCCCTAACTATCA | CTTA | 21 |
| R(△crtYf-41) | agccTGATAGTTAGGGAACATAGGG |  |  |
| F(△crtYf-42) | agatCCCTATGTTCCCTAACTATCAT | CTTA | 22 |
| R(△crtYf-42) | agccATGATAGTTAGGGAACATAGGG |  |  |
| F(△crtYf-43) | agatCCCTATGTTCCCTAACTATCATA | CTTA | 23 |
| R(△crtYf-43) | agccTATGATAGTTAGGGAACATAGGG |  |  |
| F(△crtYf-44) | agatCCCTATGTTCCCTAACTATCATAT | CTTA | 24 |
| R(△crtYf-44) | agccATATGATAGTTAGGGAACATAGGG |  |  |
| F(△crtYf-45) | agatCCCTATGTTCCCTAACTATCATATT | CTTA | 25 |
| R(△crtYf-45) | agccAATATGATAGTTAGGGAACATAGGG |  |  |
| F(△crtYf-46) | agatCAGGCAACCATAGGGCA | TTTC | 17 |
| R(△crtYf-46) | agccTGCCCTATGGTTGCCTG |  |  |
| F(△crtYf-47) | agatCAGGCAACCATAGGGCAG | TTTC | 18 |
| R(△crtYf-47) | agccCTGCCCTATGGTTGCCTG |  |  |
| F(△crtYf-48) | agatCAGGCAACCATAGGGCAGG | TTTC | 19 |
| R(△crtYf-48) | agccCCTGCCCTATGGTTGCCTG |  |  |
| F(△crtYf-49) | agatCAGGCAACCATAGGGCAGGA | TTTC | 20 |
| R(△crtYf-49) | agccTCCTGCCCTATGGTTGCCTG |  |  |
| F(△crtYf-50) | agatCAGGCAACCATAGGGCAGGAA | TTTC | 21 |
| R(△crtYf-50) | agccTTCCTGCCCTATGGTTGCCTG |  |  |
| F(△crtYf-51) | agatCAGGCAACCATAGGGCAGGAAT | TTTC | 22 |
| R(△crtYf-51) | agccATTCCTGCCCTATGGTTGCCTG |  |  |
| F(△crtYf-52) | agatCAGGCAACCATAGGGCAGGAATC | TTTC | 23 |
| R(△crtYf-52) | agccGATTCCTGCCCTATGGTTGCCTG |  |  |
| F(△crtYf-53) | agatCAGGCAACCATAGGGCAGGAATCA | TTTC | 24 |
| R(△crtYf-53) | agccTGATTCCTGCCCTATGGTTGCCTG |  |  |
| F(△crtYf-54) | agatCAGGCAACCATAGGGCAGGAATCAG | TTTC | 25 |
| R(△crtYf-54) | agccCTGATTCCTGCCCTATGGTTGCCTG |  |  |
| F(△upp-37) | agatAATCATTGCCGCCCAGA | GTTA | 17 |
| R(△upp-37) | agccTCTGGGCGGCAATGATT |  |  |
| F(△upp-38) | agatAATCATTGCCGCCCAGAA | GTTA | 18 |
| R(△upp-38) | agccTTCTGGGCGGCAATGATT |  |  |
| F(△upp-39) | agatAATCATTGCCGCCCAGAAG | GTTA | 19 |
| R(△upp-39) | agccCTTCTGGGCGGCAATGATT |  |  |
| F(△upp-40) | agatAATCATTGCCGCCCAGAAGA | GTTA | 20 |
| R(△upp-40) | agccTCTTCTGGGCGGCAATGATT |  |  |
| F(△upp-41) | agatAATCATTGCCGCCCAGAAGAA | GTTA | 21 |
| R(△upp-41) | agccTTCTTCTGGGCGGCAATGATT |  |  |
| F(△upp-42) | agatAATCATTGCCGCCCAGAAGAAG | GTTA | 22 |
| R(△upp-42) | agccCTTCTTCTGGGCGGCAATGATT |  |  |
| F(△upp-43) | agatAATCATTGCCGCCCAGAAGAAGA | GTTA | 23 |
| R(△upp-43) | agccTCTTCTTCTGGGCGGCAATGATT |  |  |
| F(△upp-44) | agatAATCATTGCCGCCCAGAAGAAGAC | GTTA | 24 |
| R(△upp-44) | agccGTCTTCTTCTGGGCGGCAATGATT |  |  |
| F(△upp-45) | agatAATCATTGCCGCCCAGAAGAAGACC | GTTA | 25 |
| R(△upp-45) | agccGGTCTTCTTCTGGGCGGCAATGATT |  |  |
| F(△upp-46) | agatCGTGCAGCAGCCAACGA | TTTC | 17 |
| R(△upp-46) | agccTCGTTGGCTGCTGCACG |  |  |
| F(△upp-47) | agatCGTGCAGCAGCCAACGAC | TTTC | 18 |
| R(△upp-47) | agccGTCGTTGGCTGCTGCACG |  |  |
| F(△upp-48) | agatCGTGCAGCAGCCAACGACC | TTTC | 19 |
| R(△upp-48) | agccGGTCGTTGGCTGCTGCACG |  |  |
| F(△upp-49) | agatCGTGCAGCAGCCAACGACCT | TTTC | 20 |
| R(△upp-49) | agccAGGTCGTTGGCTGCTGCACG |  |  |
| F(△upp-50) | agatCGTGCAGCAGCCAACGACCTC | TTTC | 21 |
| R(△upp-50) | agccGAGGTCGTTGGCTGCTGCACG |  |  |
| F(△upp-51) | agatCGTGCAGCAGCCAACGACCTCG | TTTC | 22 |
| R(△upp-51) | agccCGAGGTCGTTGGCTGCTGCACG |  |  |
| F(△upp-52) | agatCGTGCAGCAGCCAACGACCTCGG | TTTC | 23 |
| R(△upp-52) | agccCCGAGGTCGTTGGCTGCTGCACG |  |  |
| F(△upp-53) | agatCGTGCAGCAGCCAACGACCTCGGC | TTTC | 24 |
| R(△upp-53) | agccGCCGAGGTCGTTGGCTGCTGCACG |  |  |
| F(△upp-54) | agatCGTGCAGCAGCCAACGACCTCGGCG | TTTC | 25 |
| R(△upp-54) | agccCGCCGAGGTCGTTGGCTGCTGCACG |  |  |
| F(△crtYf-55) | agatAGCCATAATGCTGCTGC | TTTA | 17 |
| R(△crtYf-55) | agccGCAGCAGCATTATGGCT |  |  |
| F(△crtYf-56) | agatAGCCATAATGCTGCTGCC | TTTA | 18 |
| R(△crtYf-56) | agccGGCAGCAGCATTATGGCT |  |  |
| F(△crtYf-57) | agatAGCCATAATGCTGCTGCCG | TTTA | 19 |
| R(△crtYf-57) | agccCGGCAGCAGCATTATGGCT |  |  |
| F(△crtYf-58) | agatAGCCATAATGCTGCTGCCGA | TTTA | 20 |
| R(△crtYf-58) | agccTCGGCAGCAGCATTATGGCT |  |  |
| F(△crtYf-59) | agatAGCCATAATGCTGCTGCCGAG | TTTA | 21 |
| R(△crtYf-59) | agccCTCGGCAGCAGCATTATGGCT |  |  |
| F(△crtYf-60) | agatAGCCATAATGCTGCTGCCGAGG | TTTA | 22 |
| R(△crtYf-60) | agccCCTCGGCAGCAGCATTATGGCT |  |  |
| F(△crtYf-61) | agatAGCCATAATGCTGCTGCCGAGGT | TTTA | 23 |
| R(△crtYf-61) | agccACCTCGGCAGCAGCATTATGGCT |  |  |
| F(△crtYf-62) | agatAGCCATAATGCTGCTGCCGAGGTA | TTTA | 24 |
| R(△crtYf-62) | agccTACCTCGGCAGCAGCATTATGGCT |  |  |
| F(△crtYf-63) | agatAGCCATAATGCTGCTGCCGAGGTAA | TTTA | 25 |
| R(△crtYf-63) | agccTTACCTCGGCAGCAGCATTATGGCT |  |  |
| F(△crtYf-64) | agatTATTTTTATAAGCATTC | CTTA | 17 |
| R(△crtYf-64) | agccGAATGCTTATAAAAATA |  |  |
| F(△crtYf-65) | agatTATTTTTATAAGCATTCC | CTTA | 18 |
| R(△crtYf-65) | agccGGAATGCTTATAAAAATA |  |  |
| F(△crtYf-66) | agatTATTTTTATAAGCATTCCT | CTTA | 19 |
| R(△crtYf-66) | agccAGGAATGCTTATAAAAATA |  |  |
| F(△crtYf-67) | agatTATTTTTATAAGCATTCCTT | CTTA | 20 |
| R(△crtYf-67) | agccAAGGAATGCTTATAAAAATA |  |  |
| F(△crtYf-68) | agatTATTTTTATAAGCATTCCTTT | CTTA | 21 |
| R(△crtYf-68) | agccAAAGGAATGCTTATAAAAATA |  |  |
| F(△crtYf-69) | agatTATTTTTATAAGCATTCCTTTT | CTTA | 22 |
| R(△crtYf-69) | agccAAAAGGAATGCTTATAAAAATA |  |  |
| F(△crtYf-70) | agatTATTTTTATAAGCATTCCTTTTT | CTTA | 23 |
| R(△crtYf-70) | agccAAAAAGGAATGCTTATAAAAATA |  |  |
| F(△crtYf-71) | agatTATTTTTATAAGCATTCCTTTTTT | CTTA | 24 |
| R(△crtYf-71) | agccAAAAAAGGAATGCTTATAAAAATA |  |  |
| F(△crtYf-72) | agatTATTTTTATAAGCATTCCTTTTTTA | CTTA | 25 |
| R(△crtYf-72) | agccTAAAAAAGGAATGCTTATAAAAATA |  |  |
| F(△upp-55) | agatAAGGTCGATGTTTCGCG | TTTA | 17 |
| R(△upp-55) | agccCGCGAAACATCGACCTT |  |  |
| F(△upp-56) | agatAAGGTCGATGTTTCGCGG | TTTA | 18 |
| R(△upp-56) | agccCCGCGAAACATCGACCTT |  |  |
| F(△upp-57) | agatAAGGTCGATGTTTCGCGGA | TTTA | 19 |
| R(△upp-57) | agccTCCGCGAAACATCGACCTT |  |  |
| F(△upp-58) | agatAAGGTCGATGTTTCGCGGAC | TTTA | 20 |
| R(△upp-58) | agccGTCCGCGAAACATCGACCTT |  |  |
| F(△upp-59) | agatAAGGTCGATGTTTCGCGGACC | TTTA | 21 |
| R(△upp-59) | agccGGTCCGCGAAACATCGACCTT |  |  |
| F(△upp-60) | agatAAGGTCGATGTTTCGCGGACCG | TTTA | 22 |
| R(△upp-60) | agccCGGTCCGCGAAACATCGACCTT |  |  |
| F(△upp-61) | agatAAGGTCGATGTTTCGCGGACCGT | TTTA | 23 |
| R(△upp-61) | agccACGGTCCGCGAAACATCGACCTT |  |  |
| F(△upp-62) | agatAAGGTCGATGTTTCGCGGACCGTA | TTTA | 24 |
| R(△upp-62) | agccTACGGTCCGCGAAACATCGACCTT |  |  |
| F(△upp-63) | agatAAGGTCGATGTTTCGCGGACCGTAG | TTTA | 25 |
| R(△upp-63) | agccCTACGGTCCGCGAAACATCGACCTT |  |  |
| F(△upp-64) | agatGAAACTTTTAAAGGTCG | CTTA | 17 |
| R(△upp-64) | agccCGACCTTTAAAAGTTTC |  |  |
| F(△upp-65) | agatGAAACTTTTAAAGGTCGA | CTTA | 18 |
| R(△upp-65) | agccTCGACCTTTAAAAGTTTC |  |  |
| F(△upp-66) | agatGAAACTTTTAAAGGTCGAT | CTTA | 19 |
| R(△upp-66) | agccATCGACCTTTAAAAGTTTC |  |  |
| F(△upp-67) | agatGAAACTTTTAAAGGTCGATG | CTTA | 20 |
| R(△upp-67) | agccCATCGACCTTTAAAAGTTTC |  |  |
| F(△upp-68) | agatGAAACTTTTAAAGGTCGATGT | CTTA | 21 |
| R(△upp-68) | agccACATCGACCTTTAAAAGTTTC |  |  |
| F(△upp-69) | agatGAAACTTTTAAAGGTCGATGTT | CTTA | 22 |
| R(△upp-69) | agccAACATCGACCTTTAAAAGTTTC |  |  |
| F(△upp-70) | agatGAAACTTTTAAAGGTCGATGTTT | CTTA | 23 |
| R(△upp-70) | agccAAACATCGACCTTTAAAAGTTTC |  |  |
| F(△upp-71) | agatGAAACTTTTAAAGGTCGATGTTTC | CTTA | 24 |
| R(△upp-71) | agccGAAACATCGACCTTTAAAAGTTTC |  |  |
| F(△upp-72) | agatGAAACTTTTAAAGGTCGATGTTTCG | CTTA | 25 |
| R(△upp-72) | agccCGAAACATCGACCTTTAAAAGTTTC |  |  |

**Table S3**

Sequence of crRNA expression cassette used in this study

| **Sequence of gRNA expression cassette** |
| --- |
| ttgacagctagctcagtcctaggtataatggatccgaatttctactgttgtagatATGCGCAGGTGAAACACCTGCATTTggctgttttggcggatgagagaagattttcagcctgatacagattaaatcagaacgcagaagcggtctgataaaacagaatttgcctggcggcagtagcgcggtggtcccacctgaccccatgccgaactcagaagtgaaacgccgtagcgccgatggtagtgtggggtctccccatgcgagagtagggaactgccaggcatcaaataaaacgaaaggctcagtcgaaagactgggcctttcgttttatctgttgtttgtcggtgaacgctctcctgagtaggacaaatccgccgggagcggatttgaacgttgcgaagcaacggcccggagggtggcgggcaggacgcccgccataaactgccaggcatcaaattaagcagaaggccatcctgacggatggcctttttgcgtttctacaaactct |
